# A family of oxychloride amorphous solid electrolytes for long-cycling all-solid-state lithium batteries

**DOI:** 10.1038/s41467-023-39197-8

**Published:** 2023-06-24

**Authors:** Shumin Zhang, Feipeng Zhao, Jiatang Chen, Jiamin Fu, Jing Luo, Sandamini H. Alahakoon, Lo-Yueh Chang, Renfei Feng, Mohsen Shakouri, Jianwen Liang, Yang Zhao, Xiaona Li, Le He, Yining Huang, Tsun-Kong Sham, Xueliang Sun

**Affiliations:** 1 Department of Mechanical and Materials Engineering, University of Western Ontario, London, ON N6A 5B9 Canada; 2 Department of Chemistry, University of Western Ontario, London, ON N6A 5B7 Canada; 3 National Synchrotron Radiation Research Centre, 101 Hsin-Ann Road, Hsinchu, 30076 Taiwan; 4 Canadian Light Source Inc., University of Saskatchewan, Saskatoon, Saskatchewan S7N 2V3 Canada; 5 Institute of Functional Nano & Soft Materials (FUNSOM), Jiangsu Key Laboratory for Carbon-Based Functional Materials & Devices, Soochow University, Suzhou, PR China

**Keywords:** Batteries, Batteries, Batteries

## Abstract

Solid electrolyte is vital to ensure all-solid-state batteries with improved safety, long cyclability, and feasibility at different temperatures. Herein, we report a new family of amorphous solid electrolytes, xLi_2_O-MCl_y_ (M = Ta or Hf, 0.8 ≤ x ≤ 2, y = 5 or 4). xLi_2_O-MCl_y_ amorphous solid electrolytes can achieve desirable ionic conductivities up to 6.6 × 10^−3 ^S cm^−1^ at 25 °C, which is one of the highest values among all the reported amorphous solid electrolytes and comparable to those of the popular crystalline ones. The mixed-anion structural models of xLi_2_O-MCl_y_ amorphous SEs are well established and correlated to the ionic conductivities. It is found that the oxygen-jointed anion networks with abundant terminal chlorines in xLi_2_O-MCl_y_ amorphous solid electrolytes play an important role for the fast Li-ion conduction. More importantly, all-solid-state batteries using the amorphous solid electrolytes show excellent electrochemical performance at both 25 °C and −10 °C. Long cycle life (more than 2400 times of charging and discharging) can be achieved for all-solid-state batteries using the xLi_2_O-TaCl_5_ amorphous solid electrolyte at 400 mA g^−1^, demonstrating vast application prospects of the oxychloride amorphous solid electrolytes.

## Abstract (web summary)

Developing amorphous solid electrolytes for solid state lithium batteries is challenging due to limited understanding of disordered structures. Here, the authors report a family of oxychloride amorphous solid electrolytes with high ionic conductivities and promising electrochemical characteristics.

## Introduction

Along with the fast growing market of rechargeable electric vehicles (REVs), the development of all-solid-state batteries (ASSBs) is of high expectation due to their promises of safety, reliability, and high energy density^1,2^. A key component for ASSBs is solid electrolyte (SE) which can potentially enable the use of high-voltage cathodes and Li metal anode to boost the energy density^3,4^. One of the essential requirements for a favorable SE is high ionic conductivity. Crystalline SEs with long-range ordered structures have shown continuous and fast Li-ion conduction. For example, representative sulfide-based SEs, such as Li Argyodites^5,6^ and Li_10_GeP_2_S_12_ (LGPS)-type^7,8^, exhibit attractive ionic conductivities in the order of 10^−2 ^S cm^−1^. Other types of crystalline SEs including oxide-based SEs (e.g., perovskite-type^9^, sodium superionic conductor (NASICON)-type^10^, and garnet-type^11,12^) and halide-based SEs (e.g., Li-M-Cl system, M = Y, In, Sc^13–17^) also demonstrate good conductivities of 10^−4^–10^−3 ^S cm^−1^. While the ion conduction mechanisms of crystalline SEs have been widely studied to provide guidance for the search of new superionic conductors, some amorphous SEs also show good potentials but are less studied^18^.

Amorphous SEs present the primary advantages of softness, easy fabrication, low grain boundaries, wider compositional variations, and isotropic ionic conduction^19^, which are expected to compensate the drawbacks of some crystalline SEs with high grain boundary resistance, poor processibility, and high cost. Despite the diligent efforts, the research for amorphous SEs has been proceeding slowly. One major challenge is that the ionic conductivities of amorphous SEs are generally lower than those of the typical crystalline SEs. Another challenge is lacking long-range periodicity that makes it difficult to understand the ion conduction mechanism in amorphous materials. There are very limited established universal theories for structure modeling and ionic diffusivity prediction for amorphous materials^20^. Different types of reported amorphous SEs have their own advantages and disadvantages. Early researches for amorphous SEs have been reported since the 1960s^19^. The sulfide-based amorphous SEs, such as Li_2_S-P_2_S_5_^21^ and Li_2_S-SiS_2_^22^, show decent ionic conductivities around 10^−4 ^S cm^−1^, but their narrow electrochemical stability window (1.5–2.5 V vs. Li^+^/Li)^23,24^ and poor electrode compatibility^25^ significantly limit their application in ASSBs. Oxide-based amorphous SEs (such as Li_2_O-MO_x_, M = Si, B, P, Ge, etc.^26–29^, and lithium phosphorus oxynitride^30^) exhibit improved (electro)chemical stability compared to the sulfide compounds, but their poor ionic conductivities of 10^−9^–10^−6 ^S cm^−1^ at room temperature (RT, 25 °C) are far away from the benchmark (10^−3 ^S/cm) for bulk-type ASSBs^31^. Nevertheless, if amorphous SEs were to be competitive with the crystalline SEs, both high conductivity and good compatibility with favorable layered oxide cathodes are required.

Herein, we report a family of lithium-based oxychloride amorphous SEs (xLi_2_O-MCl_y_, M = Ta or Hf, 0.8 ≤ x ≤ 2, y = 5 or 4). The newly developed SEs display several desirable features compared to the existing SEs. (1) Facile synthesis method. One-step ball-milling method can easily yield the desired products in amorphous state. (2) High ionic conductivity. The optimized 1.6Li_2_O-TaCl_5_ amorphous SE possesses a high ionic conductivity of 6.6 × 10^−3 ^S cm^−1^ at 25 °C, surpassing most of the other amorphous SEs. Similarly high ionic conductivities can be maintained within an amorphous formation region (x = 1.1‒1.8) for xLi_2_O-TaCl_5_. X-ray absorption spectroscopy (XAS) and other advanced techniques are combined to clarify that Ta-centered trigonal bipyramids with rich terminal chlorines are predominant for fast Li-ion diffusion. The optimized 1.5Li_2_O-HfCl_4_ amorphous SE also exhibits a good ionic conductivity of 1.97 × 10^−3 ^S cm^−1^ at 25 °C. (3) Outstanding electrochemical performance. The xLi_2_O-MCl_y_ amorphous SEs have good compatibility with different favorable oxide cathodes without any additional cathode coatings. ASSBs using xLi_2_O-MCl_y_ amorphous SEs showed promising long-life cycling performance at both 25 °C and −10 °C.

## Results

### Preparation of the xLi_2_O-MCl_y_ materials

**Fig. 1:**
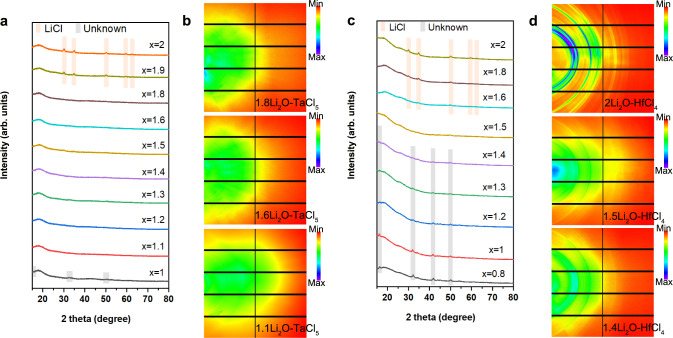
Synthesis and identification of xLi_2_O-MCl_y_ (M = Ta or Hf) amorphous materials. **a**, **c**, Lab-based XRD patterns for the as-prepared xLi_2_O-TaCl_5_ (1 ≤ x ≤ 2) (**a**) and xLi_2_O-HfCl_4_ (0.8 ≤ x ≤ 2) (**c**). The broaden peaks before 25^o^ are the diffraction peaks of the Kapton films used to seal the tested powders to avoid any air exposure. **b**, **d** Synchrotron-based 2D diffraction images of xLi_2_O-TaCl_5_ (x = 1.8, 1.6, and 1.1) (**b**) and xLi_2_O-HfCl_4_ (x = 2, 1.5, and 1.4) (**d**). Source data are provided as a Source Data file.

The xLi_2_O-TaCl_5_ amorphous SEs were prepared by simply ball-milling Li_2_O and TaCl_5_ at various stoichiometric ratios. Lab-based X-ray diffraction (XRD) patterns of the as-prepared xLi_2_O-TaCl_5_ (1 ≤ x ≤ 2) are shown in Fig. 1a. When x = 1, the pattern was generally amorphous with a few unknown impurities. Slightly increasing the molar ratio of Li_2_O/TaCl_5_ to 1.1 led to a completely amorphous feature (see zoom-in figures in Supplementary Fig. 1). Interestingly, further increase of the Li_2_O content (x ≤ 1.8) did not change the amorphous nature of xLi_2_O-TaCl_5_ SEs. As proved by synchrotron-based 2D diffraction patterns in Fig. 1b, the similar vague halos were recorded for the selected 1.1Li_2_O-TaCl_5_, 1.6Li_2_O-TaCl_5_, and 1.8Li_2_O-TaCl_5_ samples, indicating amorphous states for the xLi_2_O-TaCl_5_ SEs. Therefore, the amorphous formation region for the xLi_2_O-TaCl_5_ system was identified as 1.1 ≤ x ≤ 1.8. For a higher feeding ratio of Li_2_O (x ≥ 1.9), LiCl impurity appeared. SEM showed the continuous and compact surface morphology of one compound (1.6Li_2_O-TaCl_5_ pellet) in Supplementary Fig. 2, which was sharply contrast to other crystalline SE under the similar ball-milling conditions. In addition to the xLi_2_O-TaCl_5_, lithium-based oxychloride amorphous SEs can be extended to the other systems involving high-valence transition metal chlorides, for example, using Hf^4+^ instead of Ta^5+^. Figure 1c depicts the lab-based XRD patterns for a series of xLi_2_O-HfCl_4_ (0.8 ≤ x ≤ 2) samples. In comparison with the xLi_2_O-TaCl_5_ amorphous SEs, the formation of amorphous xLi_2_O-HfCl_4_ solids was more difficult. Only 1.5Li_2_O-HfCl_4_ exhibited relatively highest amorphous content among all the prepared xLi_2_O-HfCl_4_ (Fig. 1d). For other xLi_2_O-HfCl_4_ compositions (x ≠ 1.5), LiCl or unknown crystalline impurities could be easily observed.

### Ionic conductivity and Li-ion conduction analyses of the xLi_2_O-MCl_y_ materials

**Fig. 2:**
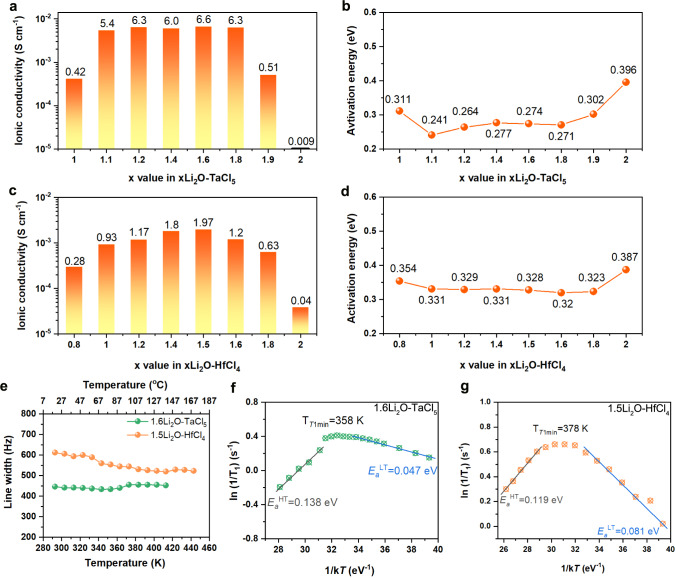
Li-ion conductivity and Li-ion diffusion behaviors of xLi_2_O-TaCl_5_ and xLi_2_O-HfCl_4_ amorphous materials. **a**, **c** The RT (25 °C) ionic conductivities for xLi_2_O-TaCl_5_ (**a**) and xLi_2_O-HfCl_4_ (**c**). **b**, **d** The corresponding activation energy values for xLi_2_O-TaCl_5_ (**b**) and xLi_2_O-HfCl_4_ (**d**) compounds. **e** The static ^7^Li motional narrowing spectra of 1.6Li_2_O-TaCl_5_ and 1.5Li_2_O-HfCl_4_. **f**, **g** Temperature-dependent ^7^Li SLR NMR rates for 1.6Li_2_O-TaCl_5_ (**f**) and 1.5Li_2_O-HfCl_4_ (**g**) measured in the laboratory frame of the reference. Source data are provided as a Source Data file.

The ionic conductivities of the new amorphous SEs were determined by measuring the electrochemical impedance spectroscopy (EIS). The xLi_2_O-TaCl_5_ (x = 1.0, 1.1, 1.2, 1.4, 1.6, 1.8, 1.9, and 2.0) powders were cold-pressed into pellets for measurements. Their temperature-dependent EIS plots are shown in Supplementary Fig. 3. Figure 2a shows the extracted conductivity values at 25 °C. When the molar ratio of Li_2_O increased from 1 to 1.1, a surge of ionic conductivity (from 0.41 × 10^−3^ to 5.3 × 10^−3 ^S cm^−1^) could be observed. Remarkably, the xLi_2_O-TaCl_5_ (1.1 ≤ x ≤ 1.8) pellets retained similarly high ionic conductivities of around 6 × 10^−3 ^S cm^−1^, which is at the top level among all other reported SEs (Supplementary Table 1). The highest ionic conductivity was 6.6 × 10^−3 ^S cm^−1^ for an optimized composition of 1.6Li_2_O-TaCl_5_. The activation energies determined from Arrhenius plots of xLi_2_O-TaCl_5_ amorphous SEs showed low values from 0.241 eV to 0.277 eV (Fig. 2b and Supplementary Fig. 4). Meanwhile, Fig. 2c compares the ionic conductivities for xLi_2_O-HfCl_4_ system (x = 0.8, 1, 1.2, 1.4, 1.5, 1.6, 1.8, and 2) at 25 °C (see corresponding Nyquist plots in Supplementary Fig. 5). The 1.5Li_2_O-HfCl_4_ SE in mostly amorphous state showed the highest ionic conductivity (1.97 × 10^−3 ^S cm^−1^) and a low activation energy (0.328 eV) among the xLi_2_O-HfCl_4_ series (Fig. 2d and Supplementary Fig. 6). Direct current (DC) measurements for the representative 1.6Li_2_O-TaCl_5_ and 1.5Li_2_O-HfCl_4_ amorphous SEs under ion-blocking and electron-blocking conditions^13,32^ were also conducted as shown in Supplementary Fig. 7. The determined electronic conductivities were negligible (at 10^−10 ^S cm^−1^ order). The Li-ion conductivities calculated from the DC measurements agree well with the values we derived from the EIS measurements, confirming the xLi_2_O-MCl_y_ amorphous SEs as excellent Li-ion conductors.

In order to analyze the Li-ion mobility in xLi_2_O-MCl_y_ amorphous SEs, solid-state nuclear magnetic resonance (SSNMR) spectroscopy was adopted to provide the nuclide-specific information on structure and dynamics. First, qualitative information on Li-ion mobility^33,34^ of the xLi_2_O-TaCl_5_ and xLi_2_O-HfCl_4_ systems was provided via ^7^Li SSNMR temperature-dependent line-shape analyses. The selected ^7^Li SSNMR spectra of the 1.6Li_2_O-TaCl_5_ and 1.5Li_2_O-HfCl_4_ samples in a temperature range of 293–443 K are displayed in Supplementary Fig. 8. For both samples, a strong resonance corresponding to the central transition (CT) of ^7^Li (I = 3/2) was observed in each spectrum. The peaks due to the CT were narrow. The linewidths (Fig. 2e) were less than 1 kHz (i.e. around 450 Hz for 1.6Li_2_O-TaCl_5_ and between 520–610 Hz for 1.5Li_2_O-HfCl_4_) without significant variations in the temperature range of 293–443 K, implying that both systems were in the extreme narrowing regime. Such observation seemed to suggest a narrow distribution of slightly different Li-ion jumping rates and, by extension, a distribution of Li diffusion pathways, which is consistent with the amorphous nature of the materials.

Second, ^7^Li NMR spin-lattice relaxation (SLR) time (T_1_) allowed us to quantitatively determine the Li-ion jumping rates and activation energies corresponding to short-range as well as long-range ion diffusions in bulk electrolytes^35–37^. Fig. 2f, g show the plots of ln (1/T_1_) versus reciprocal temperature (1/k*T*) for 1.6Li_2_O-TaCl_5_ and1.5Li_2_O-HfCl_4_, respectively. A 1/T_1_ maximum was reached for each system, corresponding to 358 and 378 K for 1.6Li_2_O-TaCl_5_ and for 1.5Li_2_O-HfCl_4_, respectively. The fact that the peak maximum of 1.6 Li_2_O-TaCl_5_ appeared at a lower temperature than 1.5Li_2_O-HfCl_4_ suggested higher Li-ion mobility in 1.6Li_2_O-TaCl_5_^38^. Li-ion jump frequency can be deduced from the maximum condition (τ·ω_0_ ≈ 1) at the relaxation rate peak^34,36,39,40^. Since the Larmor frequency of ^7^Li at 9.4 T was 155.2 MHz, the Li-ion jump frequency (1/τ) of 9.8 × 10^8 ^s^−1^ occurred at 358 and 378 K for the 1.6Li_2_O-TaCl_5_ and the 1.5Li_2_O-HfCl_4_, respectively. The activation energies, E_a_^HT^ and E_a_^LT^, derived from the slopes of high-temperature (HT) and low-temperature (LT) flanks were not equal. Specifically, for the 1.6Li_2_O-TaCl_5_ sample, the activation energies were E_a_^HT^ = 0.138 eV and E_a_^LT^ = 0.047 eV, whereas the E_a_^HT^ = 0.119 eV and E_a_^LT^ = 0.081 eV for 1.5Li_2_O-HfCl_4_. Generally, the E_a_^HT^ and E_a_^LT^ are related to by a relationship of E_a_^LT^ = (β − 1) E_a_^HT^ where 1 < β ≤ 2. For uncorrelated isotropic diffusion described by the BPP (Bloembergen, Purcell and Pound) model, E_a_^HT^ and E_a_^LT^ are equal, corresponding to β = 2^16^. In the present case, β values were determined to be 1.34 and 1.68 for the 1.6Li_2_O-TaCl_5_ and the 1.5Li_2_O-HfCl_4_ samples, respectively, indicating structurally complex Li-ion conductions^40–42^. Generally, correlation effects (e.g., Coulomb interactions, correlated ion dynamics, structural disorders, etc.) are considered closely associated with the impacted Li-ion conduction^43,44^. In our studies of ^7^Li SLR NMR for the amorphous SEs, the native structural disorder of the two amorphous samples was regarded as the major contributor towards the deviation of β value off 2 (1 <β < 2), leading to smaller E_a_^LT^ values compared to those of E_a_^HT^^33,42^. Elaboration on the relevant correlation effects of locally disordered structure on the Li-ion migration is proposed as an interesting research direction that appeals to further attention.

### Local structure exploration of xLi_2_O-MCl_y_ amorphous SEs

**Fig. 3:**
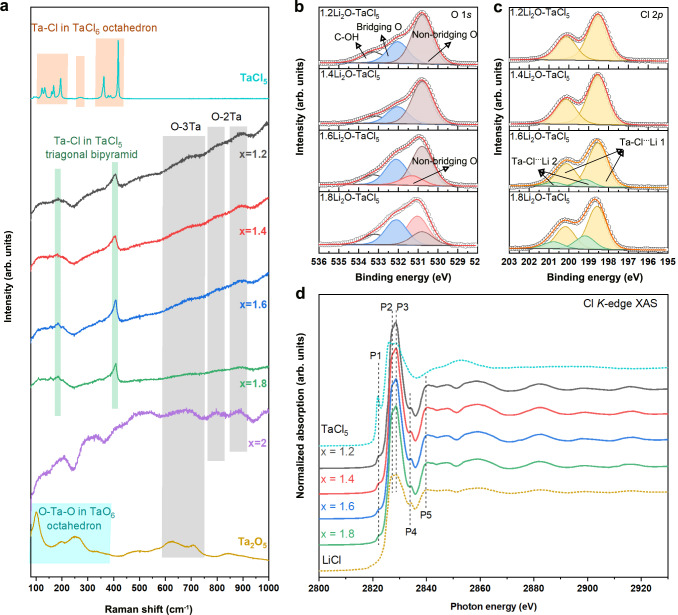
Structure characterizations and mix anion functions of xLi_2_O-TaCl_5_ amorphous SEs. **a** Raman spectra of the xLi_2_O-TaCl_5_ compounds, Ta_2_O_5_, and TaCl_5_. **b**–**d** O 1 *s* XPS (**b**), Cl 2*p* XPS (**c**), and Cl *K*-edge XAS (**d**) for xLi_2_O-TaCl_5_ amorphous SEs. Source data are provided as a Source Data file.

The Li-ion transport environment and structural information of representative xLi_2_O-TaCl_5_ were investigated by using Raman spectroscopy, X-ray Photoelectron Spectroscopy (XPS) and XAS. Figure 3a depicts the Raman spectra of xLi_2_O-TaCl_5_ (x = 1.2, 1.4, 1.6, 1.8, and 2). It was interesting to find that the bands at 180 cm^−1^ and 406 cm^−1^ for xLi_2_O-TaCl_5_ (x = 1.2, 1.4, 1.6 and 1.8) corresponded to Ta–Cl vibrations in trigonal bipyramidal TaCl_5_^45,46^, which implied a dissociation of Ta_2_Cl_10_ bi-octahedra to TaCl_5_ trigonal bipyramid when introducing a moderate amount of Li_2_O into TaCl_5_ under high-energy ball-milling conditions. In comparison with noticeable Ta–Cl features, Ta–O fingerprints in xLi_2_O-TaCl_5_ were broader to show double/triple-coordinated oxygen stretching (O-3Ta/O-2Ta) vibrations^47,48^. This was a key signal that O atoms mainly act as bridges to connect Ta-centered trigonal bipyramids in xLi_2_O-TaCl_5_ amorphous SEs. Such an observation could also be verified in O 1 *s* XPS spectra in Fig. 3b, in which bridging oxygens with a binding energy of 532.1 eV^49,50^ showed increased fraction along with the growth of the feeding Li_2_O. This was consistent with the previous reports for oxysulfide amorphous SEs, which indicated oxygens showed strong amorphous formation ability to preferentially become bridging oxygens to connect polyhedra in a short range^51^.

Since oxygens mainly contributed to form the structure of amorphous SEs, it was highly possible that chlorines took the responsibility to conduct Li ions. The chloride anion chemistry has been proved being beneficial for Li-ion migration because of a relatively large anion radius, large anion polarizability, and weak interaction with Li-ion^52^. Because of that, unsaturated Ta–Cl···Li bonds were proposed in xLi_2_O-TaCl_5_ amorphous SEs (Fig. 3c). The interactions between lithium and chlorine could be proved by Cl *K*-edge XAS. As shown in Fig. 3d, despite a pre-edge feature P1 (2822.3 eV) was indistinctly found in xLi_2_O-TaCl_5_ amorphous SEs because a mixing of Cl *p*-orbitals and Ta *d*-orbitals increased covalency^53^, the near-edge spectra of xLi_2_O-TaCl_5_ amorphous SEs (P2, P3, P4 and P5) were very similar to that of the LiCl. These peaks reflected the Cl 1 *s* electron transition process to unoccupied states and multiple scatterings, giving direct proof that Li atom was the nearest neighbor around Cl atom. Besides, it was interesting to find the oscillation in the extended range (from 2843 to 2920 eV) of each xLi_2_O-TaCl_5_ spectrum was identical with that of the LiCl, while the xLi_2_O-TaCl_5_ curves even showed a stronger amplitude than the LiCl one. This meant the Li-ion mobility environment in xLi_2_O-TaCl_5_ amorphous SEs was similar to that in LiCl but with rich Li···Cl or Cl···Li···Cl interactions.

**Fig. 4:**
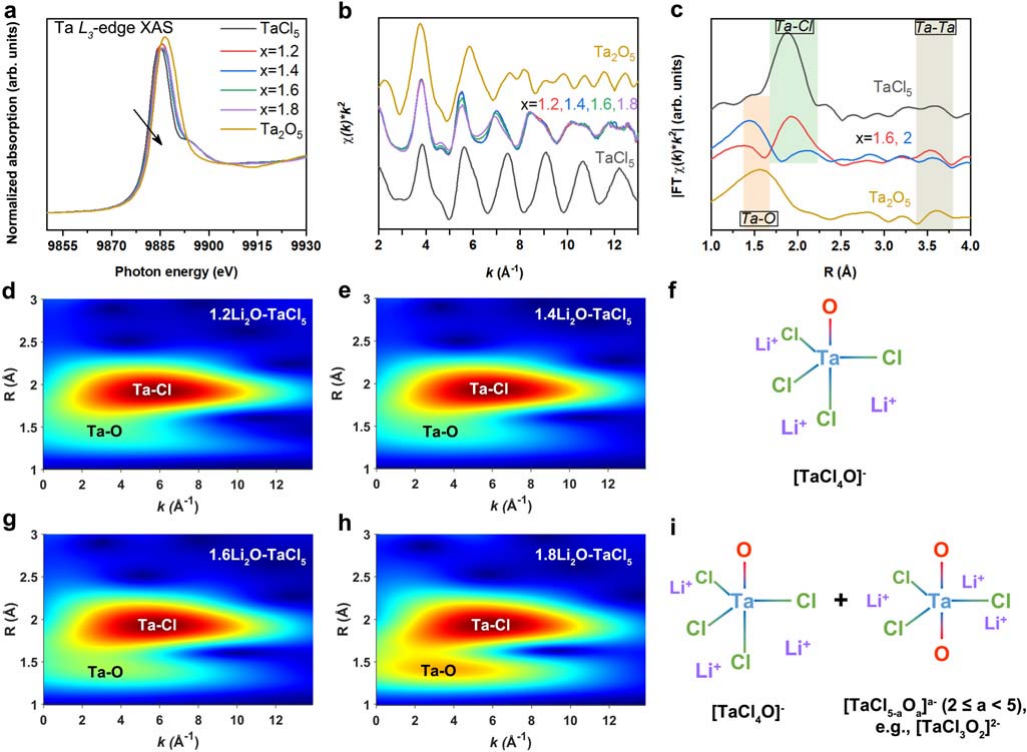
Local structure analyses of xLi_2_O-TaCl_5_ amorphous SEs. **a**–**c** XANES (**a**), EXAFS (**b**), and FT-EXAFS (**c**) for xLi_2_O-TaCl_5_ amorphous SEs, as well as Ta_2_O_5_ and TaCl_5_ at Ta *L*_*3*_-edge. **d**, **e**, **g**, **h** WT spectra of 1.2Li_2_O-TaCl_5_ (**d**), 1.4Li_2_O-TaCl_5_ (**e**), 1.6Li_2_O-TaCl_5_ (**g**), and 1.8Li_2_O-TaCl_5_ (**h**) at Ta *L*_*3*_-edge with a *k*^2^ weighting. **f**, **i** The schematics of the local structures in superionic xLi_2_O-TaCl_5_ amorphous SEs (x = 1.2 and 1.4 in (**f**); x = 1.6 and 1.8 in (**i**)). (The local structures mainly display the coordination and possible geometry of xLi_2_O-TaCl_5_ amorphous SEs. Bond angles and Li-ion numbers are not accurate.) Source data are provided as a Source Data file.

Then, the Ta *L*_3_-edge extended X-ray absorption fine structure (EXAFS) spectra were employed to determine the atomic-scale chemical environment of amorphous xLi_2_O-TaCl_5_ SEs. In the Ta *L*_3_-edge XAS spectra, since the white line (WL) at Ta *L*_3_-edge corresponds to the dipolar transition from *2p*_*3/2*_ core levels to unoccupied Ta *5d* states, the WL intensity and peak position increase when the oxidation state of Ta increases, and vice versa^54^. As depicted in Fig. 4a, the XANES spectra of xLi_2_O-TaCl_5_ (x = 1.2, 1.4, 1.6, 1.8) possessed a similar feature (9888 to 9898 eV) with that of the TaCl_5_. The absorption edge energy (E_0_) at Ta *L*_3_-edge for xLi_2_O-TaCl_5_ was between 9883 eV (Ta_2_O_5_) and 9882 eV (TaCl_5_), ascribing to the oxidation of TaCl_5_ when Li_2_O was added. Figure 4b shows Ta *L*_3_-edge EXAFS of xLi_2_O-TaCl_5_ in *k*-space. The weakened amplitude in the range of 4 to 8 Å^−1^ and a low-*k* phase shift could be observed especially for 1.6Li_2_O-TaCl_5_ and 1.8Li_2_O-TaCl_5_. This was a signal for elongation and disorder of Ta–Cl bonds caused by Ta–O bonding, which could also be reflected in the XPS results (Fig. 3c). To further resolve the coordination of Ta in xLi_2_O-TaCl_5_, phase-uncorrected radial distribution functions (RDF) after Fourier Transformed (FT) EXAFS and wavelet transformed (WT) EXAFS were conducted^55,56^. Based on the Ta–O and Ta–Cl scattering paths in referential TaCl_5_ and Ta_2_O_5_ (Supplementary Fig. 9), Ta in each xLi_2_O-TaCl_5_ amorphous SE could be recognized to be coordinated by O and Cl (Fig. 4c–e, j, h). Intensive Ta-O signals could be observed for 1.6Li_2_O-TaCl_5_ and 1.8Li_2_O-TaCl_5_. EXAFS fitting (Supplementary Fig. 10) provided a semi-quantitative explanation about these differences in xLi_2_O-TaCl_5_ amorphous SEs, and the results were listed in Supplementary Table 2. The coordination number (CN) nearest to Ta could be estimated to be 5 in each xLi_2_O-TaCl_5_ compound, consistent with the coordination situations for Ta-centered trigonal bipyramids. As a result, we could determine that the local structure in superionic 1.2Li_2_O-TaCl_5_ and 1.4Li_2_O-TaCl_5_ amorphous SEs was mainly [TaCl_4_O]^-^ trigonal bipyramid (Fig. 4f). Feeding more Li_2_O induced mixed short-order structures as [TaCl_4_O]^-^ and O-rich [TaCl_5-a_O_a_]^a-^ (2 ≤ a < 5) in 1.6Li_2_O-TaCl_5_ and 1.8Li_2_O-TaCl_5_ amorphous SEs (Fig. 4i). Besides, the connection of local structures in xLi_2_O-TaCl_5_ amorphous SEs could also be found as noticeable Ta–O–Ta (also write as Ta–Ta) resonances (12 Å^−1^) in Supplementary Fig. 11, which was of highly similarity with the Ta–O–Ta resonance in Ta_2_O_5_. Therefore, [TaCl_4_O]^-^ and [TaCl_5-a_O_a_]^a-^ shared most of the oxygens to form the networks of amorphous SEs were further confirmed.

Based on above structural information, explanations for high conductivity of xLi_2_O-TaCl_5_ amorphous SEs could be proposed as following. First, disordered and irregular [TaCl_5-a_O_a_]^a-^ (1 ≤ a < 5) arrangements in amorphous xLi_2_O-TaCl_5_ SEs made it possible to form rich Li–Cl interactions and distorted Li–Cl sublattices. Second, corner-shared oxygen (O-2Ta) networks could induce a much wider range of distortions in Li sites^57^. The distorted lithium sites in O-2Ta networks were the predominance to realize a Li-ion energy landscape with low migration energy. At the same time, oxygens at the bridging positions would enlarge the doorway radius for easy access of Li ions^58^. Third, the unsaturated Ta–Cl···Li bonds in [TaCl_5-a_O_a_]^a-^ (1 ≤ a < 5) showed weak Coulombic forces between lithium and chlorine_,_ making it easier for Li ions escape from one site and jump to another. In short, oxygen incorporation is beneficial to the amorphization of xLi_2_O-TaCl_5_. The induced disordered structures in amorphous xLi_2_O-TaCl_5_ lead to a sharply increased ionic conductivity and decreased activation energy compared to the single-anion Li-Ta-Cl sample (see comparison data in Supplementary Fig. 12).

The local structures of amorphous 1.5Li_2_O-HfCl_4_ were also explored by Hf *L*_*3*_-edge XANES and EXAFS. Similar to the behaviors in xLi_2_O-TaCl_5_, the E_0_ of Hf *L*_*3*_-edge XANES in 1.5Li_2_O-HfCl_4_ was between those of the in HfCl_4_ and HfO_2_ (Supplementary Fig. 13). Combing with the WT-EXAFS spectra (Supplementary Fig. 14), it was shown that in 1.5Li_2_O-HfCl_4_, Hf was also nearest coordinated by O and Cl (Supplementary Fig. 15 and Supplementary Table 3). The XAS of superionic conductive 1.5Li_2_O-HfCl_4_ amorphous SE and poor ionic conductor 2Li_2_O-HfCl_4_ were compared, which showed 1.5Li_2_O-HfCl_4_ amorphous SE with more Hf–Cl bonds while 2Li_2_O-HfCl_4_ with obvious Hf–O interactions (Supplementary Fig. 13c). Based on the structural information in xLi_2_O-TaCl_5_, it was reasonable to know that the formation the abundant terminal chlorines and moderate bridging oxygens in 1.5Li_2_O-HfCl_4_ amorphous SEs was predominant to the fast Li-ion conduction.

### Electrochemical performance of ASSBs

**Fig. 5:**
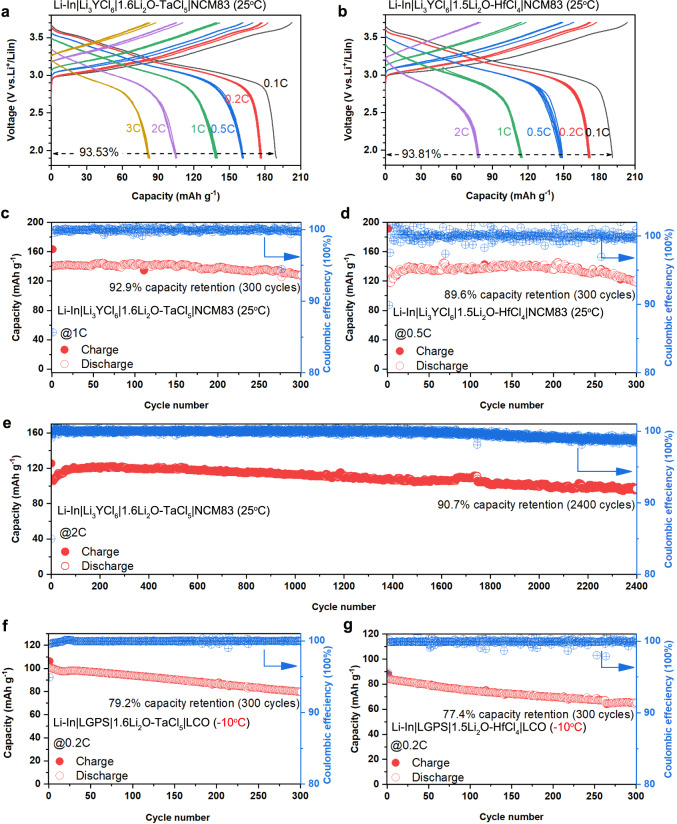
Electrochemical performances of ASSBs using 1.6Li_2_O-TaCl_5_ or 1.5Li_2_O-HfCl_4_ amorphous SEs. **a**, **b** Charge–discharge curves extracted from rate performance measurements of NCM83 ASSBs at room temperature (RT, 25 °C) with 1.6Li_2_O-TaCl_5_ (**a**) and1.5Li_2_O-HfCl_4_ (**b**). **c**–**e** RT cycling performances of NCM83 ASSBs with 1.6Li_2_O-TaCl_5_ (**c**) and 1.5Li_2_O-HfCl_4_ (**d**), and long-life cycling of NCM83 ASSB using 1.6Li_2_O-TaCl_5_ at 2 C (**e**). **f**, **g** −10 °C cycling performances of LCO ASSBs using 1.6Li_2_O-TaCl_5_ (**f**) and 1.5Li_2_O-HfCl_4_ (**g**). Note: The specific currents of 1 C for NCM83 and LCO cathode materials are corresponding to 200 mA g^−1^ and 140 mA g^−1^, respectively. Source data are provided as a Source Data file.

As promising SEs for bulk-type all-solid-state batteries (ASSBs), the electrochemical stability windows of 1.6Li_2_O-TaCl_5_ and 1.5Li_2_O-HfCl_4_ amorphous SEs were measured (Supplementary Fig. 16). Although the amorphous SEs were thermodynamically unstable against metal anodes (such as Li and Li-In) due to the reduction of Ta^5+^ or Hf^4+^, they showed good oxidative stability and are promising to tolerate cathode active materials beyond 4 V. The electrochemical performances of 1.6Li_2_O-TaCl_5_ and 1.5Li_2_O-HfCl_4_ amorphous SEs were then evaluated with LiNi_0.83_Co_0.11_Mn_0.06_O_2_ (NCM83) and LiCoO_2_ (LCO). The NCM83 ASSB with 1.6Li_2_O-TaCl_5_ SE exhibited good rate performance as shown in Fig. 5a. The charge-discharge curves depict high reversible capacities of 189.5 mAh g^−1^, 177.1 mAh g^−1^, 161.7 mAh g^−1^, 138 mAh g^−1^, 105.7 mAh g^−1^ and 83 mAh g^−1^ at 0.1 C, 0.2 C, 0.5 C, 1 C, 2 C, and 3 C, respectively (1 C = 200 mA g^−1^). As for the NCM83 ASSB with 1.5Li_2_O-HfCl_4_ SE, the rate performance was comparable with the 1.6Li_2_O-TaCl_5_ cell at lower rates (191.1 mAh g^−1^ @ 0.1 C, 171.9 mAh g^−1^ @ 0.2 C, 149.2 mAh g^−1^ @ 0.5 C) (Fig. 5b). NCM83 ASSBs integrated with either amorphous SE showed superior long cycle life at designated specific currents. As shown in Fig. 5c, the ASSB with 1.6Li_2_O-TaCl_5_ SE demonstrated highly stable cycling performance for over 300 cycles with a capacity retention of 92.9% at 1 C. The 1.5Li_2_O-HfCl_4_ ASSB also retained 89.6% of its initial reversible capacity after 300 cycles at 0.5 C (Fig. 5d). In particular, the impressive cycling durability at 2 C was demonstrated for the solid cell after evaluation of its rate capability (Fig. 5a). As depicted in Fig. 5e, there was a capacity retention of 90.7% after 2400 cycles. The charge-discharge curves suggested effective electrochemical reversibility during the long-cycling measurement (Supplementary Fig. 17). Due to the high ionic conductivity of 1.6Li_2_O-TaCl_5_, we further examined the ASSBs at a low temperature of −10 °C. The LCO ASSB exhibited an initial reversible capacity of 100.6 mAh g^−1^ with a Coulombic efficiency (CE) of 94.9%. The cell maintained a high capacity of 93.7 mAh g^−1^ after 100 cycles and stably operated for over 300 cycles with a capacity retention of 79.2% (average CE: 99.94%) (Fig. 5f). Meanwhile, despite that the 1.5Li_2_O-HfCl_4_ SE possessed a lower ionic conductivity of 3.4 × 10^−4 ^S cm^−1^ at −10 °C, the LCO solid cell with 1.5Li_2_O-HfCl_4_ could still stably cycle over 300 cycles at 0.2 C (1 C = 140 mA g^−1^) (Fig. 5g**)**. The rate performance of LCO ASSBs at −10 °C was also decent with either Ta-based or Hf-based amorphous SE (Supplementary Fig. 18). Finally, in order to demonstrate the practical prospect of amorphous SEs, 1.6Li_2_O-TaCl_5_ was chosen to be applied in pouch cell, which exhibited decent cycling performance in Supplementary Fig. 19.

In summary, we report a series of new amorphous superionic conductors, xLi_2_O-TaCl_5_ (x = 1.1‒1.8) and xLi_2_O-HfCl_5_ (x = 1.5), which can be prepared via a one-step ball-milling method. Among them, the optimized 1.6Li_2_O-TaCl_5_ and 1.5Li_2_O-HfCl_4_ amorphous SEs possess high ionic conductivities of 6.6 × 10^−3 ^S cm^−1^ and 1.97 × 10^−3 ^S cm^−1^, respectively, at 25 °C. The local environment in the representative superionic Ta-based amorphous SEs is identified as [TaCl_5-a_O_a_]^a-^ (1 ≤ a < 5) trigonal bipyramids in which abundant terminal chlorines directly interact with Li ions weakly. Bridging oxygens that serve as joints in the networks of amorphous SEs can induce a wide range of distortions in Li sites. Fast Li-ion conduction in xLi_2_O-TaCl_5_ amorphous SEs benefits from such a mixed anion chemistry. The 1.6Li_2_O-TaCl_5_ and 1.5Li_2_O-HfCl_4_ amorphous SEs also show good cathode compatibility with conventional layered oxide cathode materials (NCM83 and LCO), performing outstanding electrochemical performances at both 25 °C and −10 °C. This study shall provide insights into the Li-ion dynamics and design principles of amorphous SEs, leading to a key advancement for ASSBs.

## Methods

### Synthesis of xLi_2_O-MCly (M = Ta or Hf) solid electrolytes

TaCl_5_ (Sigma Aldrich, 99.99%), HfCl_4_ (Sigma Aldrich, 98%), LiCl (Sigma Aldrich, reagent grade), and Li_2_O (Alfa Aesar, 99.5%) were used as the raw materials. The starting materials for each compound were mixed in an argon-filled glovebox (H_2_O < 0.1 ppm, O_2_ < 0.1 ppm). The resulting mixture (1 g) was then placed in a zirconia ball milling pot along with 40 g zirconia balls. Low-speed ball milling (100 rpm for 2 h) was first run to ensure all the precursors mixed well, followed by a high-speed ball milling process of 500 rpm for 10 h. Next, the ball-milled products were transferred into the glovebox for further use. Similarly, the Li_3_YCl_6_ electrolyte was prepared by ball milling YCl_*3*_ and LiCl in mole ratio of 1: 3. The starting materials (1 g) were weighted and pre-mixed in an argon-filled glovebox, which then placed in a zirconia ball milling pot along with 40 g zirconia balls. Low-speed ball milling (100 rpm for 2 h) was first run to ensure all the precursors mixed well, followed by a high-speed ball milling process of 500 rpm for 10 h.

### Measurements of ionic and electronic conductivities

Ionic conductivity of as-prepared SEs was evaluated using electrochemical impedance spectroscopy (EIS) with two stainless steel rods as blocking electrodes. The SE powders (100~150 mg) were cold-pressed into pellets under ~300 MPa. The thickness of the pellets was between 0.04 cm and 0.06 cm. EIS measurements were performed using a multichannel potentiostat 3/Z (German VMP3). The applied frequency range was 1 Hz‒7 MHz and the voltage amplitude was 20 mV. The temperature control was realized in an ESPEC Environmental Test Chamber. The cell assembly process for DC measurements was similar with that for EIS tests. To determine the electronic conductivity, the current responses of the cell was measured at a range of constant voltages for 60 min each. The applied voltage ranged from 0.1 to 0.5 V with a step size of 0.1 V. The DC Li-ion conductivity was evaluated with a symmetric cell configuration of Li|Li_6_PS_5_Cl | xLi_2_O-MCl_y_ | Li_6_PS_5_Cl | Li under a bias voltage for 30 min. The bias voltage was applied at 5, 10, 15, 20, and 25 mV consecutively. The Li_6_PS_5_Cl SE (provided by China Automotive Battery Research Institute Co, Ltd) was used to prevent direct contact between Li metal and xLi_2_O-MCl_y_ SE.

### Linear sweep voltammetry (LSV) test

Approximately 80 mg of the 1.6Li_2_O-TaCl_5_ or 1.5Li_2_O-HfCl_4_ amorphous SE powder was cold-pressed into a pellet. A 10-mg mixture of amorphous SE and carbon black (CB) (8:2 wt./wt.) was uniformly covered on one side of the pellet as working electrode. Li foil was attached on the other side of the pellet as both counter and reference electrode. A Li_6_PS_5_Cl interlayer (~40 mg) was adopted to avoid the incompatibility between amorphous SE and metallic Li. The LSV measurements were conducted using a versatile multichannel potentiostat 3/Z (VMP3) with a positive scan range from open-circuit voltage (OCV) to 6 V and a negative scan range from OCV to 0 V. The scan rate was 0.1 mV s^−1^.

### Assembly and electrochemical characterizations of ASSBs

60 mg of 1.6Li_2_O-TaCl_5_ and 1.5Li_2_O-HfCl_4_ amorphous SEs were pressed at ~300 MPa to form a solid electrolyte layer (10 mm diameter), respectively. 10 mg of amorphous SE/NCM83 composite (3:7 mass ratio) was uniformly spread onto the surface of the one side of electrolyte layer and pressed with ~360 MPa for 5 min. NCM83 (LiNi_0.83_Co_0.11_Mn_0.06_O_2_) cathode material (polycrystalline particle size: ~3 μm) was provided by China Automotive Battery Research Institute Co, Ltd. Subsequently, Li-In alloy was placed on the other side of the electrolyte layer and pressed by ~120 MPa for 3 min. The Li-In alloy was prepared by pressing a piece of In foil (ϕ 10 mm, thickness 0.1 mm) and a piece of Li foil (ϕ 10 mm, thickness 20 μm) together under ~60 MPa for 5 min. To prevent the direct contact between amorphous SE and Li-In. 40 mg of Li_3_YCl_6_ powder pressed into pellet was served as the interlayer at the anode side. The obtained internal pellet cell was sandwiched between two stainless-steel rods as current collectors. Finally, a stack pressure of ~80 MPa was applied to the solid cell for various electrochemical tests. All cell fabrication processes were carried out in an Ar-filled glove box (H_2_O, O_2_ < 0.1 ppm). For the −10 °C full-cell test, the assemble process was similar as above. However, we changed the anode electrolyte to Li_10_GeP_2_S_12_ (LGPS) to provide a high ionic conductivity at −10 °C. LGPS was purchased from MSE Supplies LLC, showing a high ionic conductivity around 6 × 10^−3 ^S cm^−1^ at 25 °C with cold-pressed pellet. LCO was used as cathode material. The specific currents of 1 C for NCM83 and LCO cathode materials are corresponding to 200 mA g^−1^ and 140 mA g^−1^, respectively. The electrochemical performances were evaluated using the Neware and Land battery testing system. The temperature of 25 °C (RT) for the battery testing was realized in a designated battery testing lab equipped with a temperature control system. The battery tests under the temperature of −10 °C were realized by a freezer manufactured by Thermo Fisher Scientific. Prior to the tests, all cells were rest and equilibrated for 12 h to reach target temperatures.

### Fabrication of all-solid-state pouch cell

The all-solid-state pouch cell was fabricated by stacking layers of NCM83/1.6Li_2_O-TaCl_5_ cathode, SE separators (Li_3_YCl_6_ and 1.6Li_2_O-TaCl_5_), and Li-In alloy anode. The membranes of cathode composites and SEs were made by dry-film processing method^59^, where 0.5 wt% Polytetrafluoroethylene (PTFE) were added to induce the formation of doughs and followed by calendaring to the target thickness (~80 um). The loading of the cathode was 13.125 mg cm^−2^. Stacking each layers was completed in the Ar-filled glovebox, which was then sealed in plastic vacuum bag for transferring to a dry room for further packing in the aluminum-plastic bag. A pressure of ~10 MPa was applied on the pouch cell during the cycling performance test using Neware battery testing system.

### Characterization methods

Lab-based XRD measurements were performed on Bruker AXS D8 Advance with Cu Kα radiation (λ = 1.5406 Å). Kapton tape was used to cover the sample holder to prevent from the air exposure.

Raman spectra were measured with a HORIBA Scientific LabRAM HR Raman spectrometer operated under laser beam at 532 nm. Electrolyte powders were attached on a carbon tape and covered by a transparent cover glass for the test.

^7^Li SSNMR SLR measurements were performed on a Varian Infinity Plus wide-bore NMR spectrometer equipped with an Oxford wide-bore magnet (**B**_0_ = 9.4 T). The ^7^Li Larmor frequency was 155.248 MHz. The π/2 and π pulse length were determined to be 2.3 and 4.5 μs, respectively. Chemical shifts were referenced with respect to a 1.0 M LiCl solution. The electrolyte sample was sealed in custom-made Teflon tubes (ɸ = 4.7 mm) in an argon-filled glovebox (H_2_O < 0.1 ppm, O_2_ < 0.1 ppm). The ^7^Li spin-lattice relaxation times (T_1_) at different temperatures were determined using an inversion-recovery NMR experiment. The testing temperature ranges from 293–443 K.

Ta L_3_-edge and Hf L_3_-edge XAS data were collected at the 44 A beamline of Taiwan Photon Source (TPS) of the National Synchrotron Radiation Research Center (NSRRC) in Taiwan. The spectra were recorded in transmission mode. Cl K-edge XAS (FY mode) were collected at the SXRMB beamline at Canadian Light Source (CLS). The above data were processed with Athena and Artemis softwares. Synchrotron-based 2D XRD images were collected at VESPERS beamline at CLS. The 2D diffraction data were recorded on a Pilatus 1 M detector with a photon energy of 13 keV (λ = 0.9537 Å). Profex and ALBULA softwares were used to process the data.

XPS were collected at Surface Science Western (SSW) in Canada by using Krotos AXIS Ultra Spectrometer system. Monochromatic Al K(alpha) source was adopted. There was a specially designed inert transfer vessel allowing for SEs samples in a glove box, and transferring to the instrument without air exposure. High resolution analyses were carried out with an analysis area of 100 microns and a pass energy of 40 eV.

## Supplementary information

Supplementary Information

Peer Review File

## Data Availability

All data that support the findings of this study are provided within the paper and its Supplementary Information. All additional information is available from the corresponding authors upon request. Source data are provided with this paper.

## Source data

Source Data

## References

[1] Janek J, Zeier WG (2016). A solid future for battery development. Nat. Energy.

[2] Schmuch R, Wagner R, Horpel G, Placke T, Winter M (2018). Performance and cost of materials for lithium-based rechargeable automotive batteries. Nat. Energy.

[3] Manthiram A, Yu XW, Wang SF (2017). Lithium battery chemistries enabled by solid-state electrolytes. Nat. Rev. Mater..

[4] Zhao, W. J., Yi, J., He, P. & Zhou, H. S. Solid-state electrolytes for lithium-ion batteries: fundamentals, challenges and perspectives. *Electrochem. Energy Rev*. **5**, 574–605 (2022).

[5] Adeli P (2019). Boosting solid-state diffusivity and conductivity in lithium superionic argyrodites by halide substitution. Angew. Chem. Int. Ed..

[6] Zhou LD, Assoud A, Zhang Q, Wu XH, Nazar LF (2019). New family of argyrodite thioantimonate lithium superionic conductors. J. Am. Chem. Soc..

[7] Kamaya N (2011). A lithium superionic conductor. Nat. Mater..

[8] Kato Y (2016). High-power all-solid-state batteries using sulfide superionic conductors. Nat. Energy.

[9] Inaguma Y (1993). High ionic-conductivity in lithium lanthanum titanate. Solid State Commun..

[10] Luo JY, Xia YY (2007). Aqueous lithium-ion battery LiTi_2_(PO_4_)_3_/LiMn_2_O_4_ with high power and energy densities as well as superior cycling stability. Adv. Funct. Mater..

[11] Wu JF (2017). Gallium-doped Li_7_La_3_Zr_2_O_12_ garnet-type electrolytes with high lithium-ion conductivity. ACS Appl. Mater. Interfaces.

[12] Bernuy-Lopez C (2014). Atmosphere controlled processing of Ga-substituted garnets for high Li-ion conductivity ceramics. Chem. Mater..

[13] Asano T (2018). Solid halide electrolytes with high lithium-ion conductivity for application in 4 V class bulk-type all-solid-state batteries. Adv. Mater..

[14] Li XN (2019). Water-mediated synthesis of a superionic halide solid electrolyte. Angew. Chem. Int. Ed..

[15] Zhou LD (2020). A new halospinel superionic conductor for high-voltage all solid state lithium batteries. Energy Environ. Sci..

[16] Zhou LD (2022). High areal capacity, long cycle life 4 V ceramic all-solid-state Li-ion batteries enabled by chloride solid electrolytes. Nat. Energy.

[17] Zhang SM (2021). Advanced high-voltage all-solid-state Li-ion batteries enabled by a dual-halogen solid electrolyte. Adv. Energy Mater..

[18] Grady ZA, Wilkinson CJ, Randall CA, Mauro JC (2020). Emerging role of non-crystalline electrolytes in solid-state battery research. Front. Energy Res..

[19] Chandra A, Bhatt A, Chandra A (2013). Ion conduction in superionic glassy electrolytes: an overview. J. Mater. Sci. Technol..

[20] Bunde A, Funke K, Ingram MD (1998). Ionic glasses: history and challenges. Solid State Ion-..

[21] Mizuno F, Hayashi A, Tadanaga K, Tatsumisago M (2005). New, highly ion-conductive crystals precipitated from Li_2_S-P_2_S_5_ glasses. Adv. Mater..

[22] Kondo S, Takada K, Yamamura Y (1992). New lithium ion conductors based on Li_2_S-SiS_2_ system. Solid State Ion..

[23] Zhu YZ, He XF, Mo YF (2015). Origin of outstanding stability in the lithium solid electrolyte materials: insights from thermodynamic analyses based on first-principles calculations. ACS Appl. Mater. Interfaces.

[24] Nolan AM, Zhu YZ, He XF, Bai Q, Mo YF (2018). Computation-accelerated design of materials and interfaces for all-solid-state lithium-ion batteries. Joule.

[25] Lau J (2018). Sulfide solid electrolytes for lithium battery applications. Adv. Energy Mater..

[26] Charles RJ (1963). Some structural and electrical properties of lithium silicate glasses. J. Am. Ceram. Soc..

[27] Ehrt D (2000). Structure, properties and applications of borate glasses. Glass Technol..

[28] Martin SW (1991). Ionic-conduction in phosphate-glasses. J. Am. Ceram. Soc..

[29] Murthy MK, Ip J (1964). Studies in germanium oide sstems .1. I, phase equilibria in the system Li_2_O—GeO_2_. J. Am. Ceram. Soc..

[30] Kozen AC, Pearse AJ, Lin CF, Noked M, Rubloff GW (2015). Atomic layer deposition of the solid electrolyte LiPON. Chem. Mater..

[31] Kaup K (2020). A lithium oxythioborosilicate solid electrolyte glass with superionic conductivity. Adv. Energy Mater..

[32] Kwok CY, Xu SQ, Kochetkov I, Zhou LD, Nazar LF (2023). High-performance all-solid-state Li_2_S batteries using an interfacial redox mediator. Energy Environ. Sci..

[33] Wilkening M, Heitjans P (2012). From micro to macro: access to long-range Li^+^ diffusion parameters in solids via microscopic ^6,7^Li spin-alignment echo NMR spectroscopy. Chemphyschem.

[34] Uitz M, Epp V, Bottke P, Wilkening M (2017). Ion dynamics in solid electrolytes for lithium batteries. J. Electroceram..

[35] Grey CP, Greenbaum SG (2002). Nuclear magnetic resonance studies of lithium-ion battery materials. MRS Bull..

[36] Heitjans P, Wilkening M (2009). Ion dynamics at interfaces: nuclear magnetic resonance studies. MRS Bull..

[37] Yu C (2016). Unravelling Li-ion transport from picoseconds to seconds: bulk versus Interfaces in an Argyrodite Li_6_PS_5_Cl-Li_2_S all-solid-state Li-ion battery. J. Am. Chem. Soc..

[38] Yu C (2020). Superionic conductivity in lithium argyrodite solid-state electrolyte by controlled Cl-doping. Nano Energy.

[39] Yu C (2017). Revealing the relation between the structure, Li-ion conductivity and solid-state battery performance of the argyrodite Li_6_PS_5_Br solid electrolyte. J. Mater. Chem. A.

[40] Yu C (2018). Facile synthesis toward the optimal structure-conductivity characteristics of the Argyrodite Li_6_PS_5_Cl solid-state electrolyte. ACS Appl. Mater. Interfaces.

[41] Epp V, Gun O, Deiseroth HJ, Wilkening M (2013). Highly mobile ions: low-temperature NMR directly probes extremely fast Li^+^ hopping in Argyrodite-type Li_6_PS_5_Br. J. Phys. Chem. Lett..

[42] Hanghofer I (2019). Substitutional disorder: structure and ion dynamics of the argyrodites Li_6_PS_5_Cl, Li_6_PS_5_Br and Li_6_PS_5_I. Phys. Chem. Chem. Phys..

[43] Kuhn A (2011). Li self-diffusion in garnet-type Li_7_La_3_Zr_2_O_12_ as probed directly by diffusion-induced ^7^Li spin-lattice relaxation NMR spectroscopy. Phys. Rev. B.

[44] Xu ZM, Chen X, Zhu H, Li X (2022). Anharmonic cation-anion coupling dynamics assisted lithium-ion diffusion in sulfide solid electrolytes. Adv. Mater..

[45] Huglen R, Poulsen FW, Mamantov G, Begun GM (1979). Characterization of tantalum pentachloride containing melts by Raman-spectroscopy. Inorg. Chem..

[46] Beattie, I. R. & Ozin, G. A. Gas-phase raman spectroscopy of trigonal bipyramidal pentachlorides and pentabromides. *J. Chem. Soc. A*, 1691–1693 (1969).

[47] Joseph C, Bourson P, Fontana MD (2012). Amorphous to crystalline transformation in Ta_2_O_5_ studied by Raman spectroscopy. J. Raman Spectrosc..

[48] Stavrou E, Zaug JM, Bastea S, Kunz M (2017). A study of tantalum pentoxide Ta_2_O_5_ structures up to 28GPa. J. Appl. Phys..

[49] Minami T, Hayashi A, Tatsumisago M (2000). Preparation and characterization of lithium ion-conducting oxysulfide glasses. Solid State Ion..

[50] Nesbitt HW (2011). Bridging, non-bridging and free (O^2-^) oxygen in Na_2_O-SiO_2_ glasses: an X-ray Photoelectron Spectroscopic (XPS) and nuclear magnetic resonance (NMR) study. J. Non-Cryst. Solids.

[51] Christensen R, Olson G, Martin SW (2013). Ionic conductivity of mixed glass former 0.35Na_2_O+0.65[xB_2_O_3_ + (1-x)P_2_O_5_] glasses. J. Phys. Chem. B.

[52] Liu YS, Wang S, Nolan AM, Ling C, Mo YF (2020). Tailoring the cation lattice for chloride lithium-ion conductors. Adv. Energy Mater..

[53] Li WH (2020). Unraveling the origin of moisture stability of halide solid-state electrolytes by in situ and operando synchrotron X-ray analytical techniques. Chem. Mater..

[54] Tsuchiya T (2011). X-Ray absorption, photoemission spectroscopy, and Raman scattering analysis of amorphous tantalum oxide with a large extent of oxygen nonstoichiometry. Phys. Chem. Chem. Phys..

[55] Xia ZM, Zhang H, Shen KC, Qu YQ, Jiang Z (2018). Wavelet analysis of extended X-ray absorption fine structure data: theory, application. Phys. B Condens.

[56] Munoz M, Argoul P, Farges F (2003). Continuous cauchy wavelet transform analyses of EXAFS spectra: a qualitative approach. Am. Mineral..

[57] Jun K (2022). Lithium superionic conductors with corner-sharing frameworks. Nat. Mater..

[58] Kim Y, Saienga J, Martin SW (2006). Anomalous ionic conductivity increase in Li_2_S+GeS_2_+GeO_2_ glasses. J. Phys. Chem. B.

[59] Wang CH (2022). Solvent-free approach for interweaving freestanding and ultrathin inorganic solid electrolyte membranes. ACS Energy Lett..

