# Supplementary material for: A family of oxychloride amorphous solid electrolytes for long-cycling all-solid-state lithium batteries
Source: Nat Commun. 2023 Jun 24;14:3780. doi: 10.1038/s41467-023-39197-8 (PMC10290651; doi:10.1038/s41467-023-39197-8)
Supplement: Supplementary file 1 — Supplementary Information [file 41467_2023_39197_MOESM1_ESM.pdf]

Supplementary Information for

A family of oxychloride amorphous solid electrolytes for long-cycling all-solid-state lithium batteries

Shumin Zhang[1,2†], Feipeng Zhao[1†], Jiatang Chen[2], Jiamin Fu[1,2], Jing Luo[1], Sandamini H Alahakoon[2], Lo-Yueh Chang[3], Renfei Feng[4], Mohsen Shakouri[4], Jianwen Liang[1], Yang Zhao[1], Xiaona Li[1], Le He[5], Yining Huang[2], Tsun-Kong Sham[2*], Xueliang Sun[1*]

[1] Department of Mechanical and Materials Engineering, University of Western Ontario, London, Ontario, N6A 5B9, Canada

[2] Department of Chemistry, University of Western Ontario, London, Ontario, N6A 5B7, Canada

[3] National Synchrotron Radiation Research Centre, 101 Hsin-Ann Road, Hsinchu, 30076, Taiwan

[4] Canadian Light Source Inc., University of Saskatchewan, Saskatoon, Saskatchewan, S7N 2V3, Canada

[5] Institute of Functional Nano & Soft Materials (FUNSOM), Jiangsu Key Laboratory for Carbon-Based Functional Materials & Devices, Soochow University, Suzhou, PR China

[†] These authors contribute equally: Shumin Zhang, Feipeng Zhao.

* Corresponding authors: tsham@uwo.ca; xsun9@uwo.ca

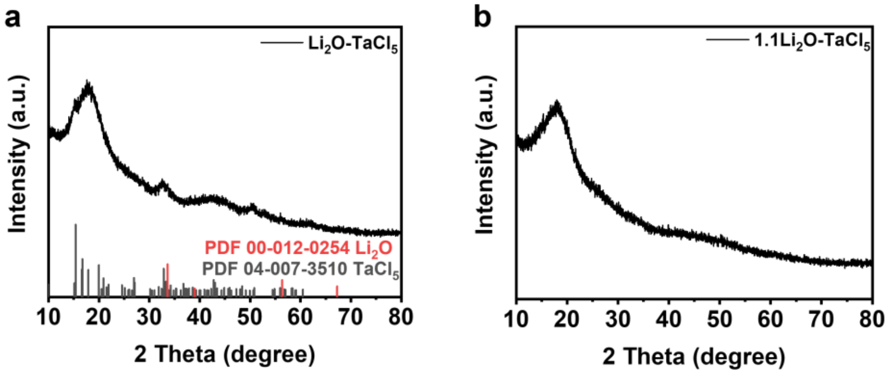

Supplementary Fig. 1 Lab-based XRD patterns for (a) Li_2O-$TaCl_5$ and (b) 1.1Li_2O-$TaCl_5$. The large peak between 10-22° is the diffraction peak of the Kapton film used to seal the tested powders to avoid any air exposure.

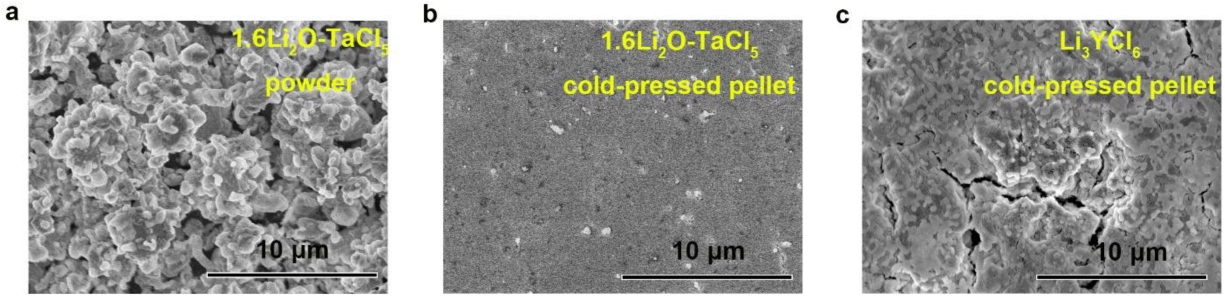

Supplementary Fig. 2 SEM images of (a) 1.6Li_2O-$TaCl_5$ as-prepared powder, (b) 1.6Li_2O-$TaCl_5$ cold-pressed pellet, and (c) crystalline Li_3YCl_6 cold-pressed pellet.

.

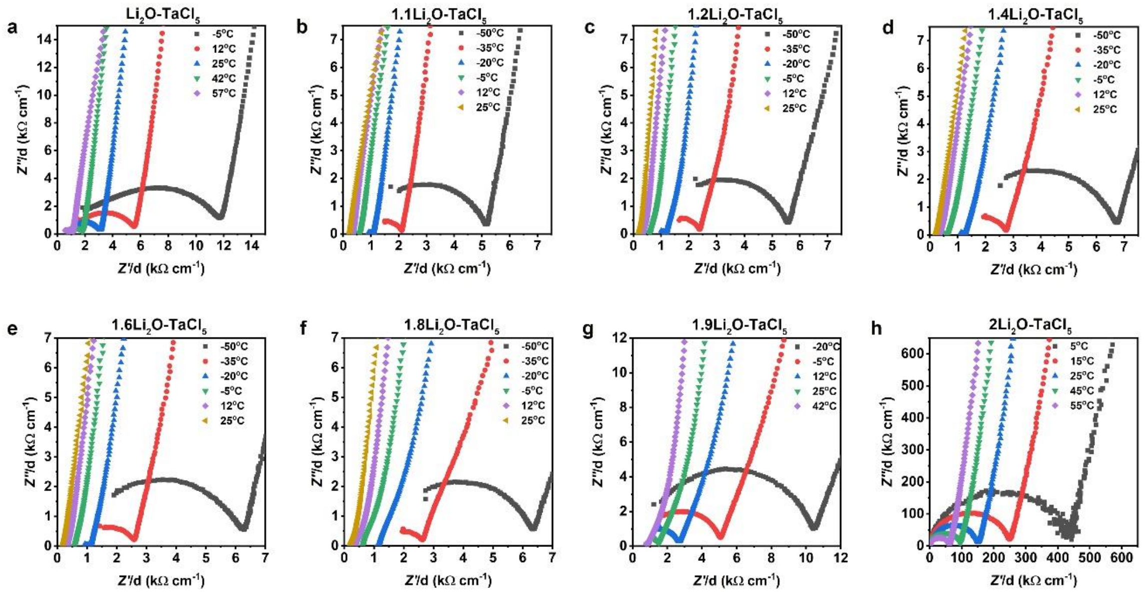

Supplementary Fig. 3 Normalized Nyquist plots for the xLi_2O-$TaCl_5$ cold-pressed pellets at various temperatures.

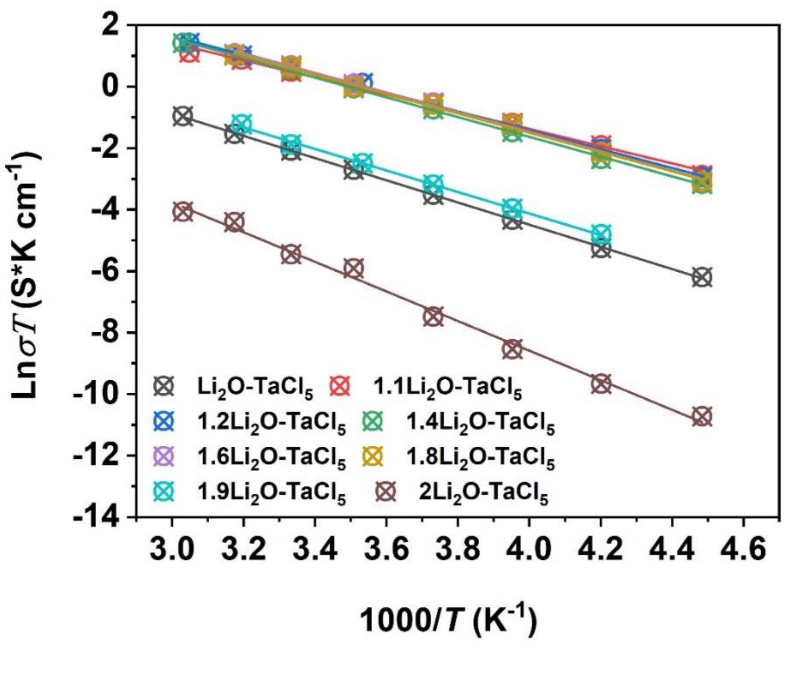

Supplementary Fig. 4 Arrhenius plots for xLi_2O-$TaCl_5$ SEs.

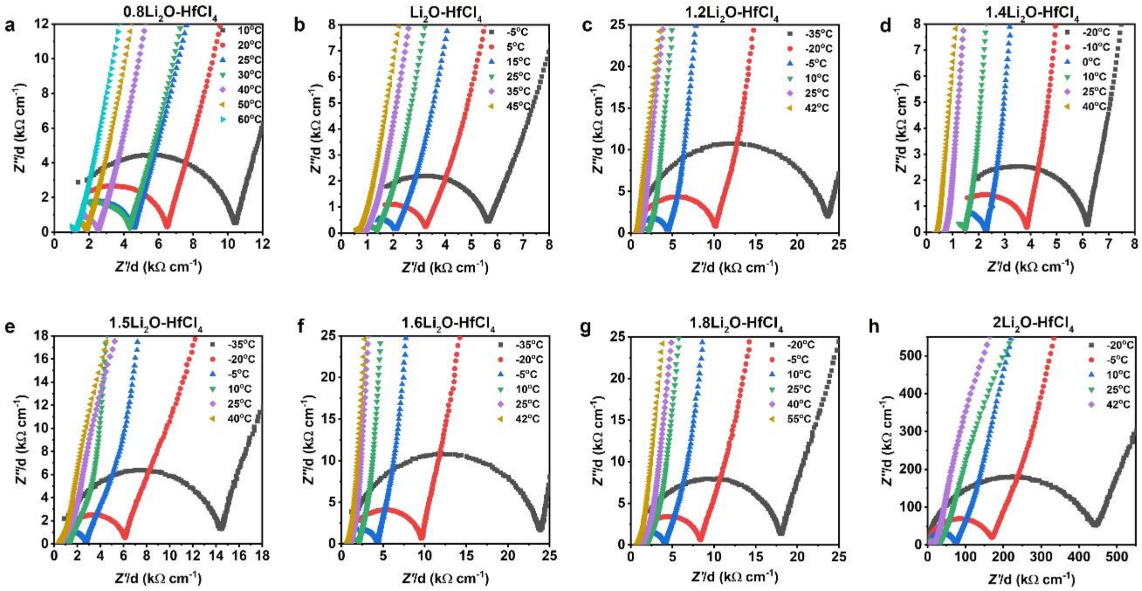

Supplementary Fig. 5 Normalized Nyquist plots for the xLi_2O-$HfCl_4$ pellets at various temperatures.

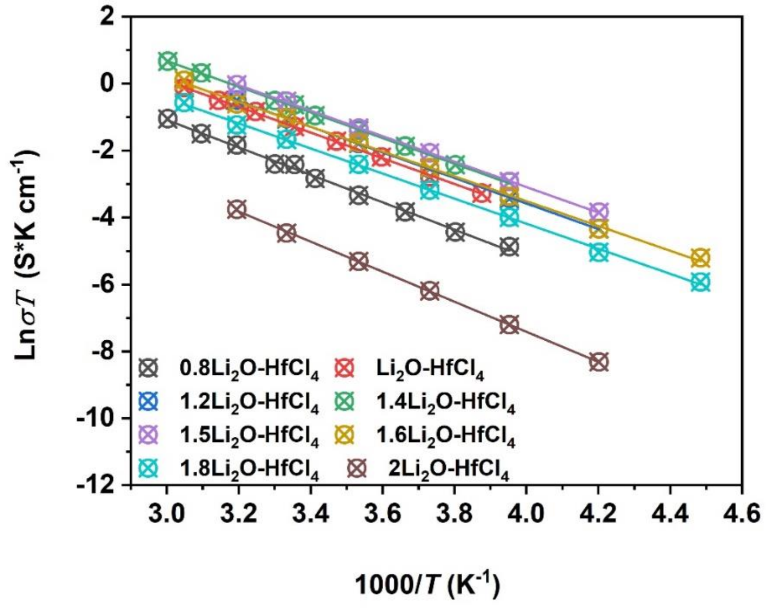

Supplementary Fig. 6 Arrhenius plots of the xLi_2O-$HfCl_4$ SEs.

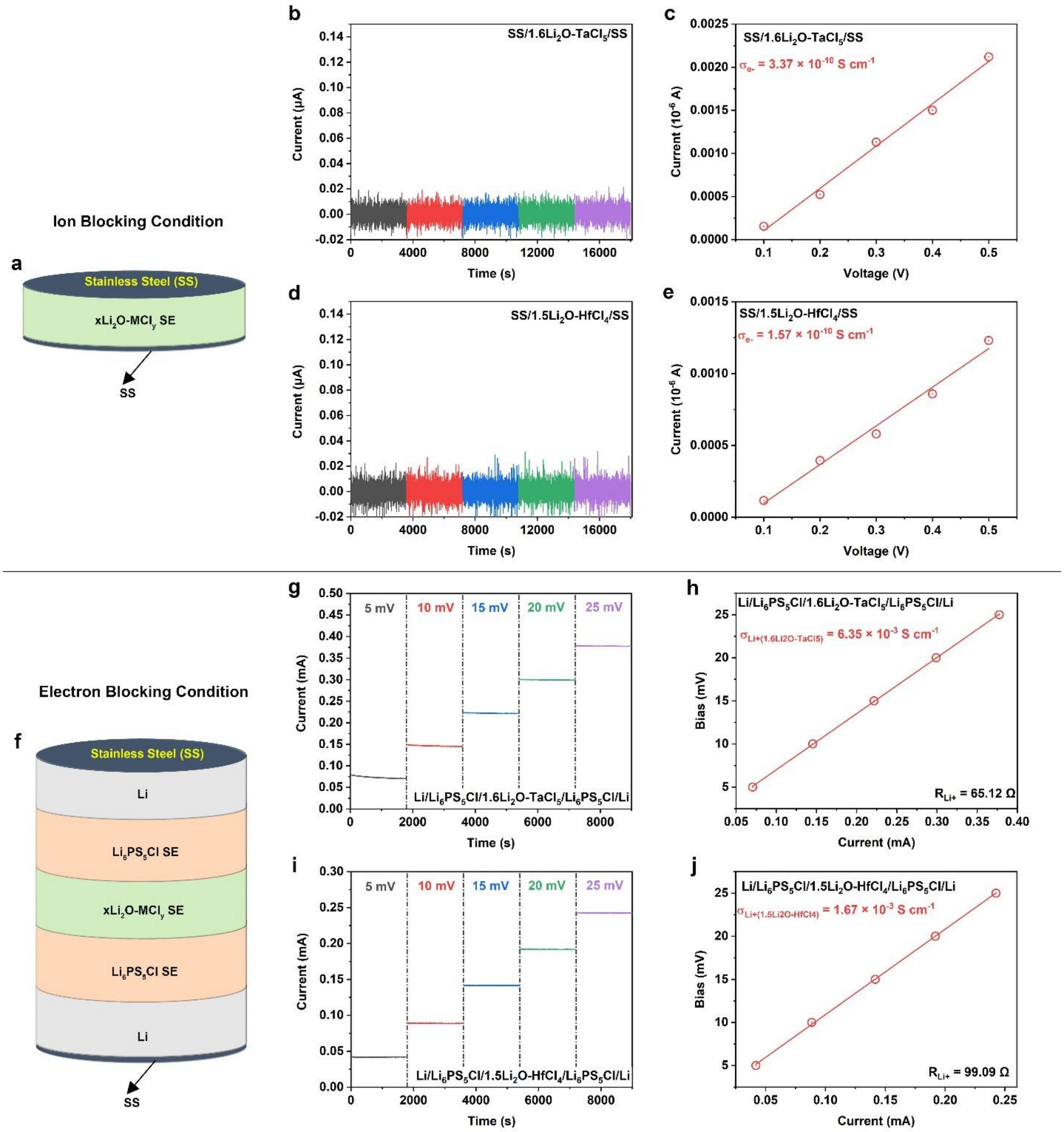

Supplementary Fig. 7 Electronic conductivity (a-e) and ionic conductivity (f-j) determination by DC measurements at room temperature. (a) Cell configuration, (b, d) DC polarization curves, and (c, e) equilibrium current response for 1.6Li_2O-$TaCl_5$ and 1.5Li_2O-$HfCl_4$ symmetric cells under ion blocking condition. (f) The electron-blocking cell configuration and (g-j) the DC polarization

results for Li-ion conductivity evaluations of 1.6Li_2O-$TaCl_5$ and 1.5Li_2O-$HfCl_4$. Li_6PS_5Cl was chosen as an interlayer SE since it is a recognized Li-ion conductive SE and kinetically stable with Li metal. The electronic conductivities of 1.6Li_2O-$TaCl_5$ and 1.5Li_2O-$HfCl_5$ were 3.37×10^{-10} S cm^{-1} and 1.57×10^{-10} S cm^{-1}, respectively. The DC measurements derived that Li-ion conductivities of 6Li_2O-$TaCl_5$ and 1.5Li_2O-$HfCl_5$ were 6.35×10^{-3} S cm^{-1} and 1.67×10^{-3} S cm^{-1}, respectively.

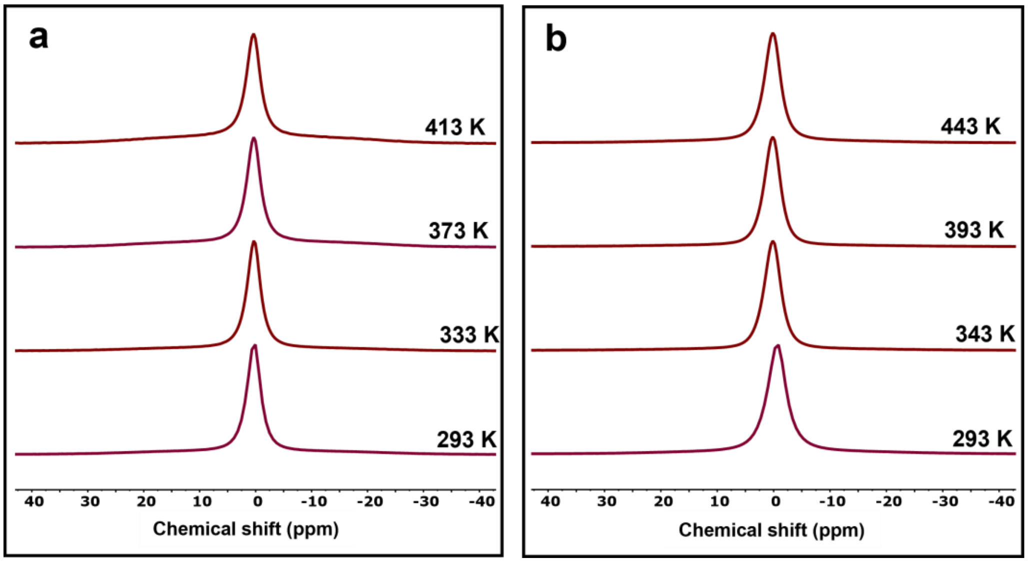

Supplementary Fig. 8 ^{7}Li SSNMR spectra of (a) 1.6Li_2O-$TaCl_5$ and (b) 1.5Li_2O-$HfCl_4$ at designated temperatures.

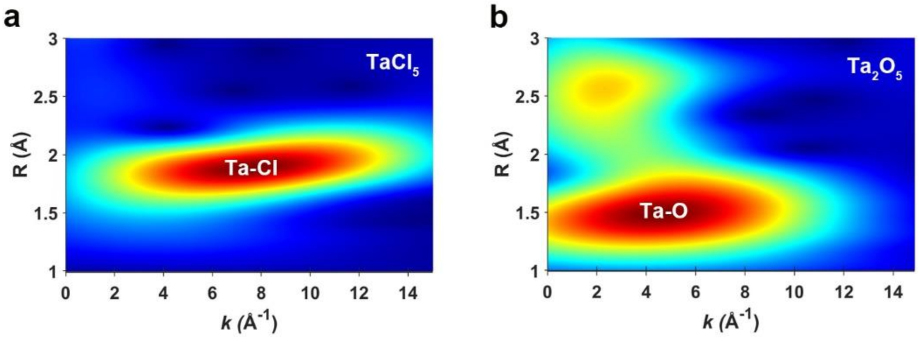

Supplementary Fig. 9 Wavelet transformed spectra of (a) $TaCl_5$ and (b) Ta_2O_5 at Ta L_3-edge with a k^2 weighting.

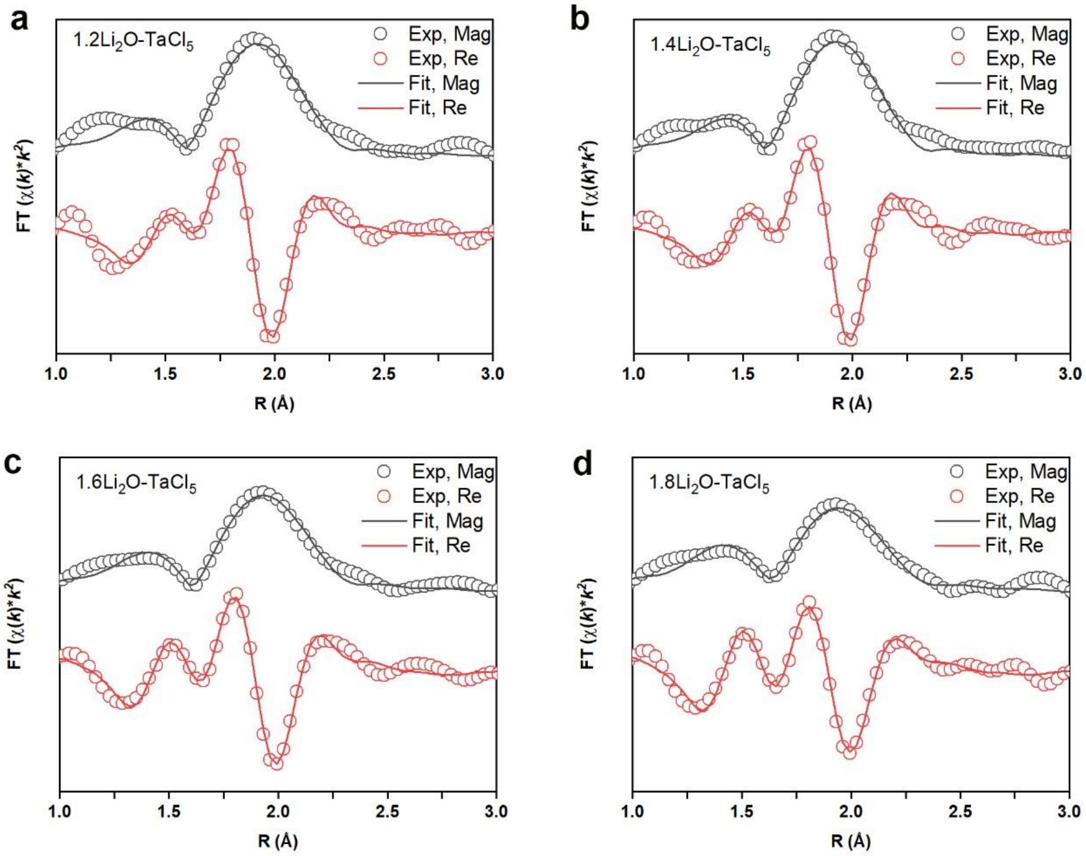

Supplementary Fig. 10 Fitting results of the k^2-weighted FT spectra of (a) 1.2Li_2O-$TaCl_5$, (b) 1.4Li_2O-$TaCl_5$, (c) 1.6Li_2O-$TaCl_5$, and (d) 1.8Li_2O-$TaCl_5$ at Ta L_3-edge, showing the experimental data (grey circle) and Feff modeling (grey line) in terms of magnitude of FT and the real part of FT experimental data (red circle) and Feff[1] modeling (red line) traces. The R factor for each fitting is 0.026, 0.023, 0.014, and 0.013, respectively. In each fitting, S_0^2 is set as 0.9114.

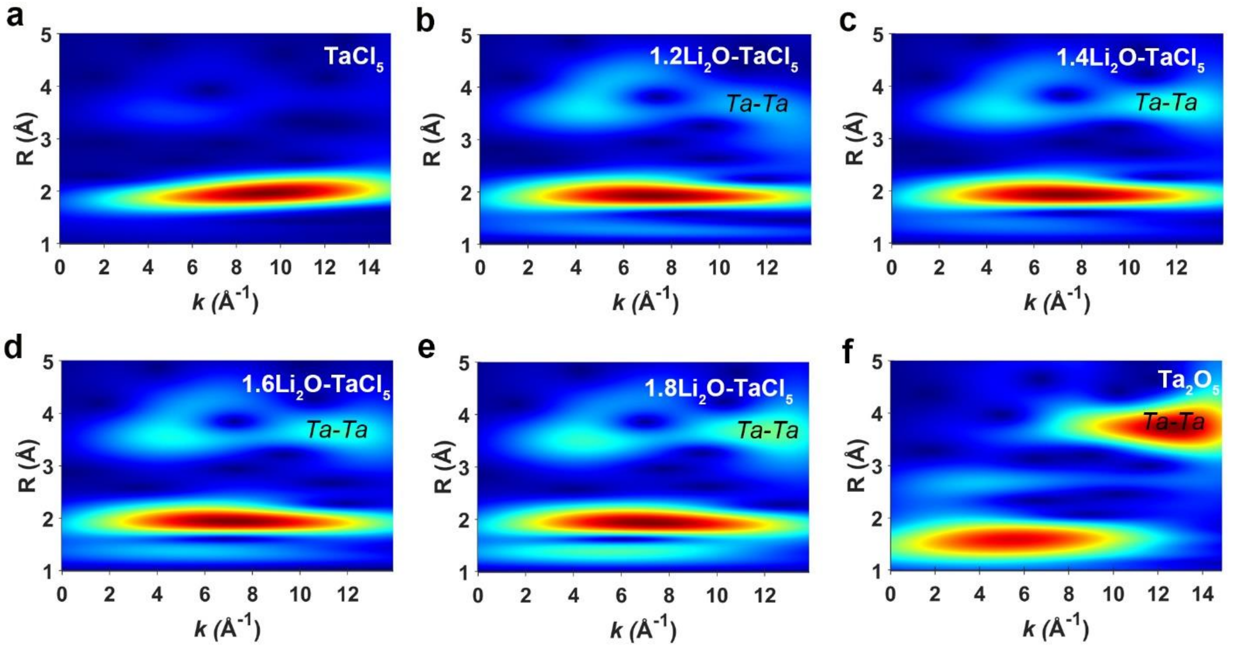

Supplementary Fig. 11 Wavelet transformed Ta L_3-EXAFS for (a) $TaCl_5$, (b) $1.2Li_2O$-$TaCl_5$, (c) $1.4Li_2O$-$TaCl_5$, (d) $1.6Li_2O$-$TaCl_5$, (e) $1.8Li_2O$-$TaCl_5$, and (f) Ta_2O_5 with a k^3 weighting.

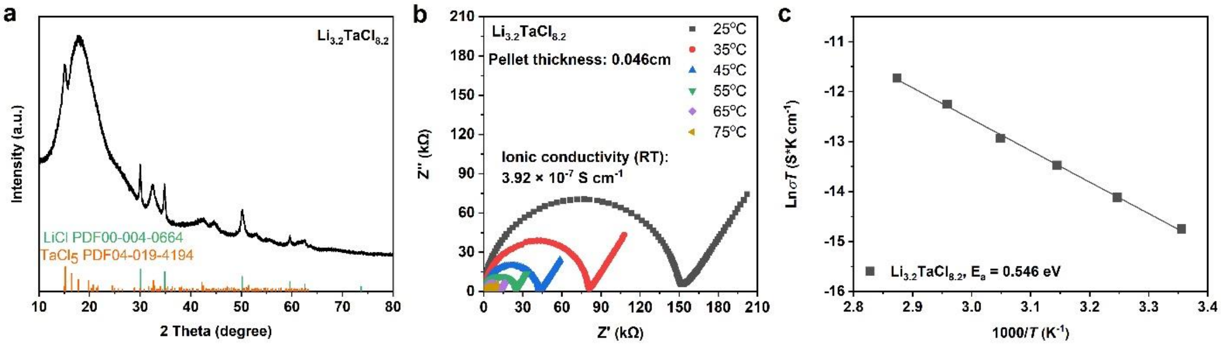

Supplementary Fig. 12 A Li-Ta-Cl sample without O incorporation was prepared following the same experimental procedure for xLi_2O-$TaCl_5$. The composition of $Li_{3.2}TaCl_{8.2}$ was chosen for the same Li/Ta ratio as the most conductive $1.6Li_2O$-$TaCl_5$ amorphous SE: (a) Lab-based XRD patterns for the as-prepared $Li_{3.2}TaCl_{8.2}$. The hump between 10° and 30° is the diffraction peak of

the Kapton film which was used to protect the sample from air exposure. The diffraction peaks can be assigned to the $TaCl_5$ and LiCl raw materials. (b) Nyquist plots and (c) Arrhenius plot for the $Li_{3.2}TaCl_{8.2}$ pellet at various temperatures. Overall, without O incorporation, the completed amorphization of Li-Ta-Cl would be difficult. The RT ionic conductivity of $Li_{3.2}TaCl_{8.2}$ dropped to the order of 10^{-7} S cm^{-1} with a significantly increased activation energy of 0.546 eV.

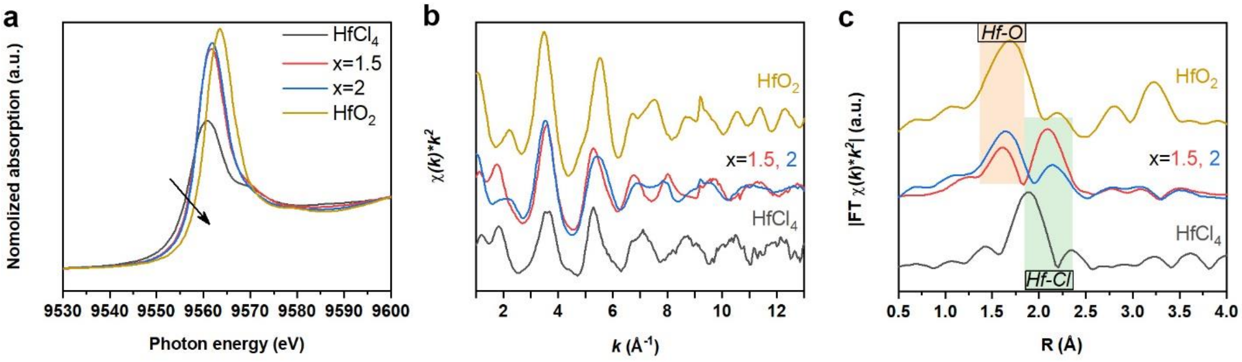

Supplementary Fig. 13 (a) XANES, (b) EXAFS, and (c) FT-EXAFS of xLi_2O-$HfCl_4$ (x = 1.5 and 2) amorphous SEs, as well as HfO_2 and $HfCl_4$ referential samples at Hf L_3-edge.

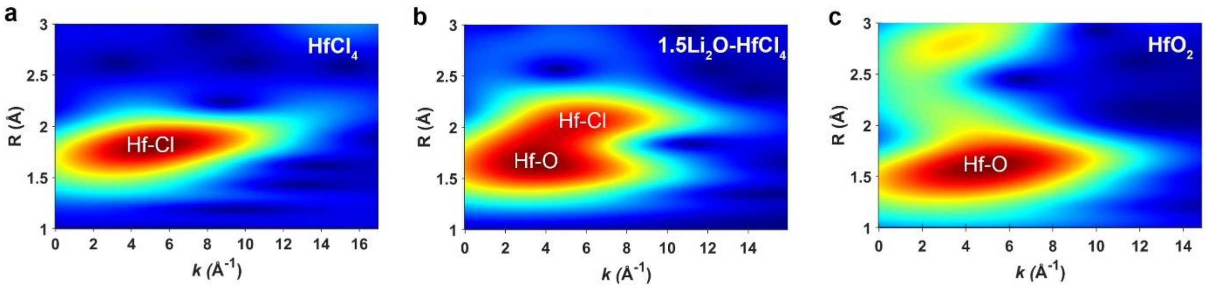

Supplementary Fig. 14 Wavelet transformed Hf L_3-EXAFS for (a) $HfCl_4$, (b) 1.5Li_2O-$HfCl_4$, (c) HfO_2 with a k^2 weighting.

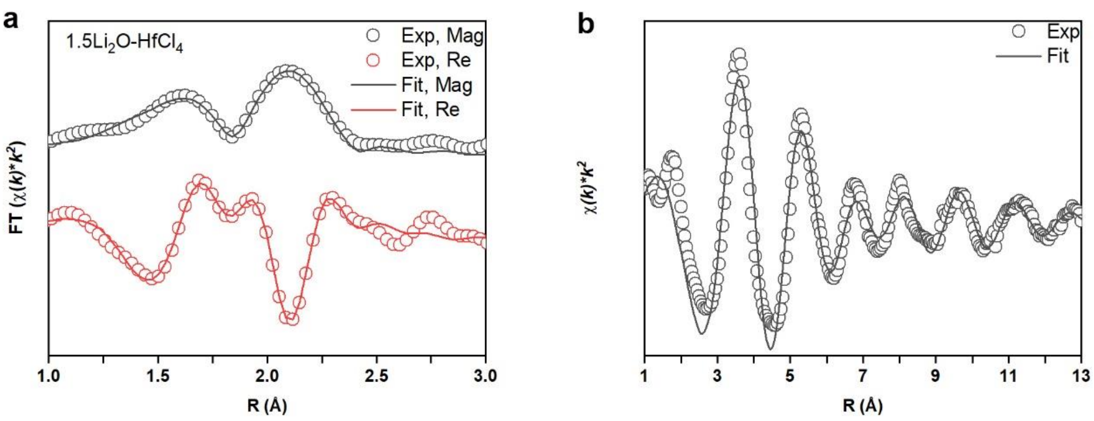

Supplementary Fig. 15 (a) Fitting results of k^2-weighted FT spectra of $1.5Li_2O\text{-}HfCl_4$ at Hf L_3-edge, showing the experimental data (grey circle) and Feff modeling (grey line) in terms of magnitude of FT and the real part of FT experimental data (red circle) and Feff[1] modeling (red line) traces. (b) Hf L_3-edge EXAFS and fit curve for $1.5Li_2O\text{-}HfCl_4$ in k-space. R factor for this fitting is 0.008. S_0^2 is set as 0.9114.

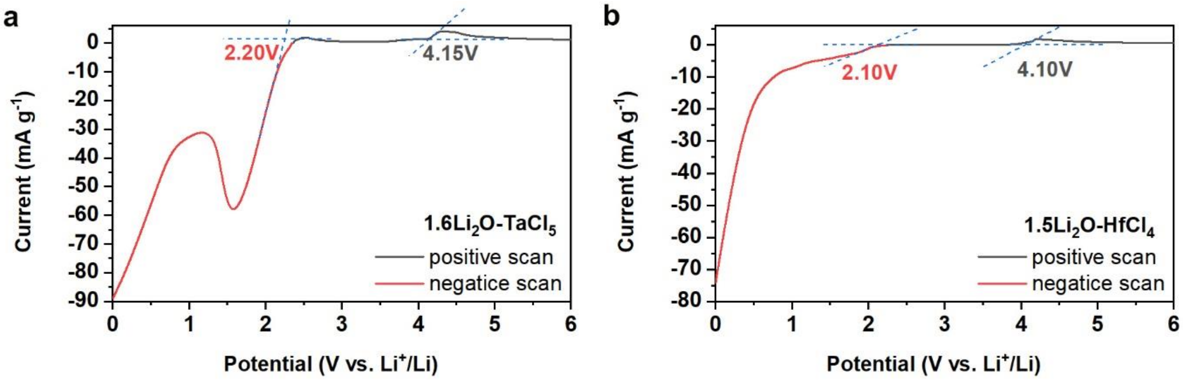

Supplementary Fig. 16 Linear cyclic voltammetry (LSV) profiles of (a) $1.6Li_2O\text{-}TaCl_5$ and (b) $1.5Li_2O\text{-}HfCl_4$.

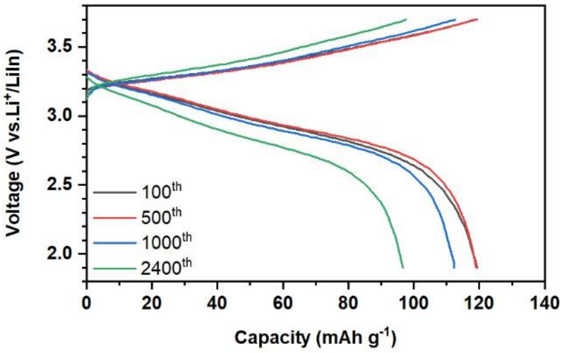

Supplementary Fig. 17 Charge-discharge curves of long-term cycling of the ASSB using $1.6Li_2O\text{-}TaCl_5$ (after rate cycling) at 2 C.

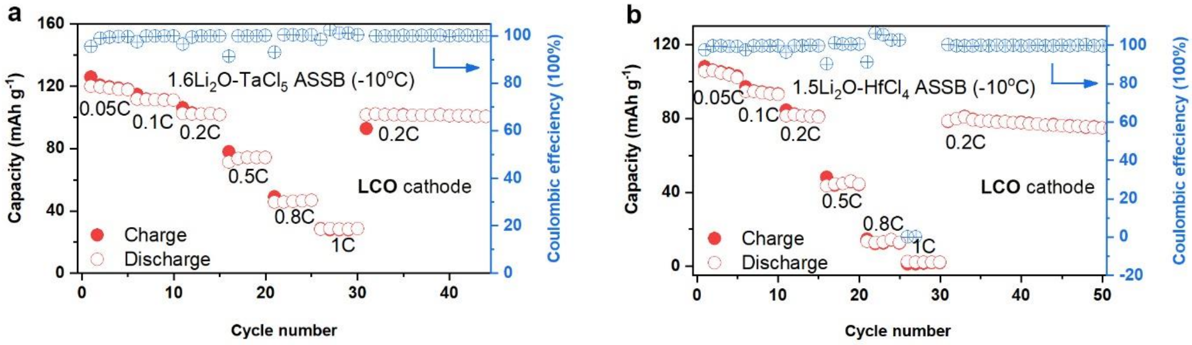

Supplementary Fig. 18 Rate performance of LCO ASSBs using (a) $1.6Li_2O\text{-}TaCl_5$ and (b) $1.5Li_2O\text{-}HfCl_4$ SEs at -10 ^oC

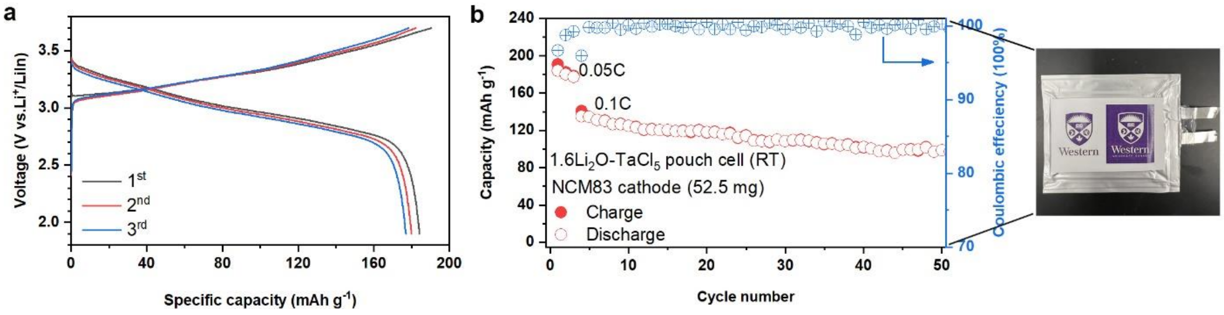

Supplementary Fig. 19 Pouch cell using $1.6Li_2O$-$TaCl_5$ SE at RT. (a) The first three charge and discharge curves in a voltage range of 1.9-3.7 V (vs. Li^+/LiIn) at 0.05 C, the initial Coulombic efficiency was 96.6%; (b) Cycling performance at 0.1 C and the photo of the pouch cell we tested.

Supplementary Table 1 Summary of the ionic conductivities of the representative SEs

Inorganic SSE category	Materials	Ionic conductivity at 25°C (S cm^{-1})	Ref.
Sulfide	$Li_{9.54}Si_{1.74}P_{1.44}S_{11.7}Cl_{0.3}$	2.5×10^{-2}	Nat. Energy 2016, 1 (4), 16030.
	$Li_{10}GeP_2S_{12}$	1.2×10^{-2}	Nat. Mater. 2011, 10 (9), 682-686.
	$Li_{6.6}Si_{0.6}Sb_{0.4}S_5I$	1.5×10^{-2}	J. Am. Chem. Soc. 2019, 141 (48), 19002-19013.
	$Li_{6.6}P_{0.4}Ge_{0.6}S_5I$	5.4×10^{-3}	J. Am. Chem. Soc. 2018, 140 (47), 16330-16339
	Li_6PS_5X (X = Cl, Br)	1.0×10^{-3}	Angew. Chem. Int. Ed. 2008, 47 (4), 755-758.
	$Li_{5.5}PS_{4.5}Cl_{1.5}$	9.4×10^{-3}	Angew. Chem. Int. Ed. 2019, 58 (26), 8681-8686
	Glass-ceramic $Li_7P_3S_{11}$	3.2×10^{-3}	Adv. Mater. 2005, 17 (7), 918-921.
Halide	Li_2ZrCl_6	8.1×10^{-4}	Nat. Commun. 2021, 12(1), 1-11
	$Li_2In_xSc_{0.666-x}Cl_4$	2×10^{-3}	Nat. Energy, 2022, https://doi.org/10.1038/s41560-021-00952-0
	Li_3YCl_6	5.1×10^{-4}	Adv. Mater. 2018, 30 (44), 1803075
	Li_3YBr_6	1.7×10^{-3}	Adv. Mater. 2018, 30 (44), 1803075
	Li_3InCl_6	2.0×10^{-3}	Angew. Chem. Int. Ed. 2019, 58 (46), 16427-16432
	Li_3ScCl_6	3.0×10^{-3}	J. Am. Chem. Soc. 2020, 142 (15), 7012-7022
	$Li_2Sc_{2/3}Cl_4$	1.5×10^{-3}	Energy Environ. Sci. 2020, 13 (7), 2056-2063

**Oxide**	$Li_7La_3Zr_2O_{12}$ system	10^{-4}~10^{-3}	Angew. Chem. Int. Ed. 2007, 46, 7778–7781; Chem. Mater. 2014, 26, 3610–3617; Chem. Mater. 2016, 28, 2384–2392; ACS Appl. Mater. Interfaces 2017, 9, 1542–1552; Chem. Mater. 2017, 29, 1769–1778;
	$LiTi_2(PO_4)_3$ system	~10^{-4}	J. Electrochem. Soc. 1990, 137, 1023-1027; Acc. Chem. Res. 1994, 27, 265–270.
	$LiGe_2(PO_4)_3$ system	~10^{-3}	J. Electrochem. Soc. 2008, 155, A915–A920; Solid State Ionics 2016, 289, 180–187.
	$Li_{3x}La_{2/3-x}TiO_3$	~10^{-4}	Solid State Commun. 1993, 86, 10, 689-693
	$100[Li_{1.5}Cr_{0.5}Ti_{1.5}(PO_4)_3]$-$5SiO_2$	2.14×10^{-2}	J. Non-Cryst. Solids 2015, 409, 120–125

Supplementary Table 2. Structural parameters of xLi_2O-$TaCl_5$ (x = 1.2, 1.4, 1.6 and 1.8) amorphous SEs extracted from Ta L_3-edge EXAFS fittings.

		1.2Li_2O-$TaCl_5$	1.4Li_2O-$TaCl_5$	1.6Li_2O-$TaCl_5$	1.8Li_2O-$TaCl_5$
Ta-O path	CN	0.9 ± 0.2	0.9 ± 0.2	1.4 ± 0.4	1.7 ± 0.4
	d (Å)	1.871 ± 0.023	1.876 ± 0.021	1.866 ± 0.013	1.867 ± 0.010
	σ^2(Å^2)	0.003 ± 0.001	0.003 ± 0.001	0.004 ± 0.003	0.004 ± 0.002
Ta-Cl path	CN	3.5 ± 0.5	3.4 ± 0.5	3.3 ± 0.4	3.0 ± 0.4
	d (Å)	2.334 ± 0.013	2.336 ± 0.011	2.336 ± 0.009	2.345 ± 0.009
	σ^2 (Å^2)	0.008 ± 0.002	0.008 ± 0.002	0.009 ± 0.002	0.009 ± 0.002
	ΔE_0 (eV)	5.08 ± 1.29	5.26 ± 1.23	4.09 ± 1.02	4.25 ± 1.00

*CN, coordination number; d (Å), bonding distance; σ^2, Debye-Waller factor; ΔE_0 is the inner potential correction. Ta-O path is from the crystal structures of Ta_2O_5 (mp-10390). Ta-Cl path from the crystal structure of $TaCl_5$ (mp-29831). The fitted k range was set to be 3–13.3 Å^{-1}, and the fitted R range was set to be 1−2.7 Å. A k^2 weighting was used.

Supplementary Table 3 EXAFS fitting of 1.5Li_2O-$HfCl_4$, giving the information of bonding distance and coordination numbers

		1.5Li_2O-$HfCl_4$
Hf-O path	CN	3.6 ± 0.7
	d (Å)	2.056 ± 0.017
	σ^2	0.009 ± 0.002
Hf-Cl path	CN	2.3 ± 0.5
	d (Å)	2.457 ± 0.009
	σ^2	0.006 ± 0.002
	ΔE_0	3.74 ± 0.83

*CN, coordination number; d (Å), bonding distance; σ^2, Debye-Waller factor; ΔE_0 is the inner potential correction. The distance for Hf-O is from the crystal structures of HfO_2 (mp-776097). The distance for Hf-Cl from the crystal structure of $HfCl_4$ (mp-29422). The fitted k range was set to be 3 – 14.035 $Å^{-1}$, and the fitted R range was set to be 1 −2.6 Å. A k^2 weighting was used.

Supplementary References:

1. Ravel, B., Newville, M. ATHENA, ARTEMIS, HEPHAESTUS: data analysis for X-ray absorption spectroscopy using IFEFFIT. *Journal of Synchrotron Radiation* **12**, 537-541 (2005).
